# Insights into the domestication of avocado and potential genetic contributors to heterodichogamy

**DOI:** 10.1093/g3journal/jkac323

**Published:** 2022-12-08

**Authors:** Edwin Solares, Abraham Morales-Cruz, Rosa Figueroa Balderas, Eric Focht, Vanessa E T M Ashworth, Skylar Wyant, Andrea Minio, Dario Cantu, Mary Lu Arpaia, Brandon S Gaut

**Affiliations:** Deptartment of Ecology and Evolutionary Biology, University of California, Irvine, Irvine, CA 92697-2525, USA; Deptartment of Ecology and Evolutionary Biology, University of California, Irvine, Irvine, CA 92697-2525, USA; Department of Viticulture and Enology, University of California, Davis, Davis, CA 95616, USA; Department of Botany and Plant Sciences, University of California, Riverside, Riverside, CA 92521, USA; Department of Botany and Plant Sciences, University of California, Riverside, Riverside, CA 92521, USA; Deptartment of Ecology and Evolutionary Biology, University of California, Irvine, Irvine, CA 92697-2525, USA; Department of Viticulture and Enology, University of California, Davis, Davis, CA 95616, USA; Department of Viticulture and Enology, University of California, Davis, Davis, CA 95616, USA; Department of Botany and Plant Sciences, University of California, Riverside, Riverside, CA 92521, USA; Deptartment of Ecology and Evolutionary Biology, University of California, Irvine, Irvine, CA 92697-2525, USA

**Keywords:** domestication, avocado, population genomics, heterodichogamy, reference genome, Plant Genetics and Genomics

## Abstract

The domestication history of the avocado (*Persea americana*) remains unclear. We created a reference genome from the Gwen varietal, which is closely related to the economically dominant Hass varietal. Our genome assembly had an N50 of 3.37 megabases, a BUSCO score of 91%, and was scaffolded with a genetic map, producing 12 pseudo-chromosomes with 49,450 genes. We used the Gwen genome as a reference to investigate population genomics, based on a sample of 34 resequenced accessions that represented the 3 botanical groups of *P. americana*. Our analyses were consistent with 3 separate domestication events; we estimated that the Mexican group diverged from the Lowland (formerly known as “West Indian”) and Guatemalan groups >1 million years ago. We also identified putative targets of selective sweeps in domestication events; within the Guatemalan group, putative candidate genes were enriched for fruit development and ripening. We also investigated divergence between heterodichogamous flowering types, providing preliminary evidence for potential candidate genes involved in pollination and floral development.

## Introduction

Avocado (*Persea americana* Mill.) is a perennial, subtropical crop that is in ever-increasing demand. In the United States, for example, per capita avocado consumption has tripled over the last 2 decades. Demand in the U.S. is met partly by domestic production but principally by imports from Mexico and elsewhere. Mexico is the largest producer, where the crop is worth an estimated $2.5 billion per year (Rendón-Anaya *et al.* 2019), but other major producers include the Dominican Republic, Peru, Brazil, Indonesia, Israel, and Kenya (http://www.fao.org/faostat/en/#data/QC/visualize). Although the popularity of avocados is primarily a 20th century phenomenon (Schaffer *et al.* 2013), they have quickly grown to be a global commodity.

Remarkably, avocado cultivation is dominated by a single variety (Hass) that represents ∼90% of cultivation worldwide (Rendón-Anaya *et al.* 2019). All Hass trees are derived clonally from a tree patented in 1935. Despite the shockingly narrow genetic base of agricultural production, avocado sensu *lato* is quite genetically diverse. Some of this diversity stems from the fact that there are 3 domesticated botanical groups (formerly called “races”): *P. americana* var. *americana* Miller [(which we will call the “Lowland” group in recognition that the previously accepted name of West Indian is inaccurate (Me and Arzate-Fernández 2010)], var. *drymifolia* (Schltdl. & Cham.) S.F. Blake (the Mexican group), and var. *guatemalensis* (L.O. Williams) Scora (the Guatemalan group) (Bergh and Ellstrand 1986). The strikingly different fruit morphologies among the groups suggest that they may have been domesticated separately, a conjecture supported by genetic data (Furnier *et al.* 1990; Ashworth and Clegg 2003; Rendón-Anaya *et al.* 2019). Further genetic and ethnobotanical data suggest that the 3 groups did not come into contact until the 16th century (Storey *et al.* 1986; Ashworth and Clegg 2003). One practical consequence of this history is that each group likely contains separate alleles and/or genes of interest for crop improvement, due to their different domestication histories. Another consequence is that hybridization between groups can produce unique allelic combinations, potentially leading to agronomically useful hybrid offspring. Hass may be, in fact, an example; although its precise breeding history is not known, genetic evidence has suggested that it is a hybrid between Guatemalan and Mexican groups (Chen *et al.* 2009; Schaffer *et al.* 2013; Rendón-Anaya *et al.* 2019).

The high demand for, and economic importance of, avocados motivates breeding efforts, but breeding remains challenging for at least 3 reasons. First, the avocado is a large tree that matures slowly [5–8 years before production (Lahav and Lavi 2009)] requiring substantial space, water, and finances (Ashworth *et al.* 2019). A second major obstacle is the reproductive system. A single tree typically produces more than 1 million flowers, of which only 0.1% or fewer yield mature fruit (Davenport 1986; Davis *et al.* 1998; Bender 2002), making controlled pollinations difficult (Chen *et al.* 2007). Finally, avocado is heterodichogamous, with 2 flowering types: A and B. Type A trees are female (receptive to pollen) in the morning of the first day and shed pollen as males in the afternoon of the following day. In contrast, type B trees are female in the afternoon of the first day and male in the morning of the next day. Although heterodichogamy is likely caused by a simple underlying genetic mechanism (Renner 2001), the system is complicated by the fact that there is some leakiness that depends on environmental cues (Davenport 1986). Heterodichogamy enhances outcrossing, but it does not ensure it. Selfing has been measured at rates from 2% to >90%, depending on the variety and environmental conditions (Torres and Bergh 1978; Degani and Gazit 1984; Davenport 1986). As a result of the complexities of the mating system, avocado breeding has historically relied on open-pollinated and inter-group hybridization, to the extent that most individual varieties lack accurate breeding records (Davis *et al.* 1998; Scora *et al.* 2002; Ashworth and Clegg 2003).

These complications argue that genomics and molecular breeding are central to the continued improvement of the avocado. Molecular markers for flowering types may be particularly useful because type B avocados are crucial for pollination but typically less productive than type A varieties (Davenport 1986). Recently, Rendón-Anaya *et al*. (2019) made an important contribution toward molecular breeding by producing draft genomes of Hass and a wild Mexican accession (*P. americana* ssp. *drymifolia*). They anchored the Hass assembly to a genetic map and ultimately produced a reference genome with 512 scaffolds and a genome size of 419 Megabases (Mb), which is less than half the expected 1C genome size of 896 Mb (Arumuganathan and Earle 1991). Using this reference, they explored the hybrid history of Hass and aspects of the evolutionary genomics of the avocado. Another Hass genome was published in 2021 (Sharma *et al.* 2021); this genome was 788 Mb, which was nearly 88% of the expected genome size, but it was not anchored into scaffolds. Still, more recently, a third study has further improved the assembly of the Hass genome, leading to resolved haplotypes (Nath *et al.* 2022). To date, however, Hass has remained the only avocado genome deciphered, and several important features of the evolutionary genomics of avocados still remain unexplored. These features include further unraveling its domestication history, using evolutionary genomic tools to identify chromosomal regions of potential agronomic interest, and focusing on genomic diversity in the context of interesting traits, like A vs B flowering types.

Here, we report the genome of the Gwen variety and use that genome as a reference for evolutionary analyses. Gwen is a grandchild of Hass with similar flavor characteristics (Witney and Martin 1995) but with higher yields and better fruit storage on the tree (Bergh and Whitsell 1982). Accordingly, Gwen has been the subject of intensive breeding efforts for 3 decades, and 1 motivation for generating a Gwen reference is to bolster these efforts. In addition, we utilized a whole-genome resequencing data set from 34 avocado accessions to address 4 sets of questions. First, what does the Gwen genome tell us about the patterns of genic hemizygosity within an avocado accession? Genomic analysis of grapevine (*Vitis vinifera*), another perennial clonally propagated crop, revealed that as many as 1 in 7 genes are hemizygous, perhaps due to structural mutations that have accrued during clonal propagation (Zhou *et al.* 2019). Is avocado similar? Second, we use the Gwen genome as a reference to explore genetic diversity within avocados, specifically to assess relationships among the 3 botanical groups and to assess the hybrid origin of well-known cultivars. This last question builds on several previous investigations of genetic diversity (Ashworth and Clegg 2003; Chen *et al.* 2008, 2009) but extends the work to a genomic scale. Third, we investigate features of avocado domestication, including demographic history, selective sweeps, and chromosomal regions of high genetic differentiation. Do the 3 groups share regions of selective sweeps, which may indicate parallel selection on genic regions associated with specific traits? Finally, we perform a preliminary investigation between the A and B flowering types, with the goal of identifying genomic regions that may contribute to heterodichogamy.

## Materials and methods

### Reference sequencing and genome assembly

Gwen (US Patent: USPP5298P 1983) is a grandchild of Hass and has been central to the University of California, Riverside breeding program. We sampled young leaf materials from a Gwen clone and isolated high molecular weight genomic DNA (gDNA) using the method of (Chin *et al.* 2016). We included a containing buffer (100 mM Tris-HCl pH 8.0, 0.35 M Sorbitol, 5 mM EDTA pH 8.0, 1% (v/v) PVP 40, 2% (v/v) of 2-mercaptoethanol) prior to cell lysis to avoid co-precipitation of polysaccharides and phenolics with DNA. DNA quality was assessed with a Nanodrop 2000 spectrophotometer (Thermo Scientific, IL, USA), by a Qubit 2.0 Fluorometer with the DNA High Sensitivity kit (Life Technologies, CA, USA), and by pulsed-field gel electrophoresis. From this, a 20 µg of high molecular weight genomic DNA was needle-sheared and used as input into the SMRTbell library preparation, using the SMRTbell Express Template Preparation V2 kit (Pacific Biosciences, CA, USA). Libraries were size selected to 22–80 Kb with the BluePippin (Sage Science, MA, USA) and sequenced on the Pacific Biosciences Sequel using P6-C4 chemistry (DNA Technology Core Facility, University of California, Davis).

We assembled SMRT reads with Canu version 2.1 (Koren *et al.* 2017), using default settings and including all reads. Once assembled, polishing was performed with 2 passes of PacBio GenomicConsensus v2.33, followed by 2 passes with Pilon v1.23 (Walker *et al.* 2014) using default parameters and 19 × coverage of Gwen short-read Illumina sequencing data. HapSolo v0.1 (Solares *et al.* 2021), which identifies and removes alternative haplotypes, was then run on the assembly with default parameters and 50,000 iterations, producing the Canu + Hapsolo (C + H) assembly. Scaffolding was based on a Gwen × Fuerte genetic map (Ashworth *et al.* 2019) by aligning the C + H assembly using NCBI BLAST v2.2.31+ (Altschul *et al.* 1990). The alignments were then ordered based on linkage group ID and cM distance, using only the alignments with the highest percent identity and evalue scores to identify contig order. When the orientation of a contig could not be determined, which was true for 112 contigs representing ∼274 Mb, it was placed in the “+” (or positive) direction and marked with an asterisk in the scaffolding annotation file (10.5281/zenodo.6392169). Where necessary, contigs were bridged using N's.

### Gene and repeat annotation

Repeat annotation was based on RepeatModeler v2.0.1 in conjunction with RepeatMasker v4.1.1. RepeatModeler (Smit 2015) was run to generate a repeat database for avocado since a database for closely related species was not available. The repeat database was built using the option BuildDatabase on the Gwen C + H assembly and then used in RepeatModeler with the LTRStruct option. RepeatMasker was run using default parameters. The Gwen genome was subsequently soft masked for repeats using Bedtools v2.29.2 (Quinlan 2014) using the maskfasta run option.

For gene annotation, we mapped RNASeq reads from previous studies (Ibarra-Laclette *et al.* 2015; Xoca-Orozco *et al.* 2017; Barbier *et al.* 2019) onto the repeat masked genome using HiSat2 version 2.2.1. The resultant BAM files were merged and indexed using Samtools v1.10 (Li *et al.* 2009; Barnett *et al.* 2011) and fed to the BRAKER v2.1.6 (Hoff *et al.* 2019)/Augustus (Stanke *et al.* 2006) v3.4.0 pipeline. BRAKER was used in default mode for RNASeq data, with an additional option for soft masked reference assemblies (−softmasking). Finally, we excluded (as possible pseudogenes) any genes with exons that overlapped annotated repeats and filtered for a specific criterion related to gene integrity (Minio *et al.* 2021).

Functional annotations and Gene Ontology analyses were performed with Blast2GO v6.01 (Conesa and Götz 2008). Genes were extracted from assemblies, based on the gene annotation gff file, and then mapped to the NCBI nonredundant protein database, SwissProt, and uniref90 databases using NCBI's blastx, blastx-fast option. A protein family search was also performed using an InterPro scan. These results were then merged into a single annotation for GO mapping and Enrichment, which was performed using Blast2GO based on default options.

Genic hemizygosity was calculated following Zhou *et al*. (2019) by (*1*) remapping raw SMRT reads to the 12 pseudo-chromosomes using NGMLR v0.2.7, (*2*) inferring structural variants by feeding the alignment file to SNIFFLES (Sedlazeck *et al.* 2018), requiring a read-coverage of at least 4 to substantiate an SV, and (*3*) and quantitating insertion–deletion events that overlapped with 20% of the coding region.

### Diversity samples, sequencing, and SNP calling

We collected leaf tissue for a total of 20 *P. americana* accessions and 3 outgroups, all of which were sampled from the South Coast Research and Extension Center in Irvine, CA (Table 1). For each sample, genomic DNA was extracted from leaf samples with the Qiagen DNeasy plant kit. Paired-end sequencing libraries were constructed with an insert size of 300 bp according to the Nextera Flex (Illumina, Inc) library preparation protocol. Libraries were sequenced on the Illumina NovaSeq with cycles to target 25 × coverage. We also used Illumina reads for 10 previously sequenced *P. americana* accessions and 1 putative outgroup (Rendón-Anaya *et al.* 2019), yielding a combined data set of 34 accessions that included 30 *P. americana* accessions and 4 putative outgroup species. Short reads from all accessions were mapped to the C + H and scaffolded assemblies using BWA, version 0.7.17 (Li and Durbin 2010), and realigned and recalibrated using GATK v3.7 (McKenna *et al.* 2010). Alignment filtering was done using BCFTools v1.10.2 (Danecek *et al.* 2021) using parameters *-s LowQual -e “%QUAL < 20 || DP > 32*”.

**Table 1.** A list of the accessions used in this study; the historical ecotype assignment follows Rounds (1950) or later sources when indicated by a footnote.

Accession, variety or species	Historical ecotype*^a^*	Admixed %*^a^*	Flowering type*^i^*	PopGen group*^j^*	Origin
069-02	M*^b^*	G: 37.61 M: 62.39 L: 0.00	N/A		Unknown
263-C	L*^b^*	G: 0.00 M: 0.00 L: 1.00	N/A	L	Hunucma, Yucatan, Mexico
Anaheim*^k^*	G	G: 93.06 M: 6.94 L: 0.00	A	G	Anaheim, California, 1910
Bacon*^k^*	M*^c^* (GxM*^d^*) G*^e^*	G: 37.69 M: 62.31 L: 0.00	B		Buena Park, California, 1928
Carlsbad*^k^*	G	G: 1.00 M: 0.00 L: 0.00	A	G	Mexico, budwood collected 1912
CH-CR-25	CR*^b^*	G: 50.01 M: 7.98 L: 42.01	N/A	W	Matapalo, Puntarenas, Costa Rica
CH-G-07	G*^b^*	G: 84.08 M: 0.00 L: 15.92	N/A	W	San Cristobal de las Casas, Chiapas, Mexico
CH-G-10	G*^b^*	G: 89.28 M: 0.00 L: 10.72	N/A	W	Olanca, Chiapas, Mexico
CH-G-11	G*^b^*	G: 89.96 M: 0.00 L: 10.04	N/A	W	Olanca, Chiapas, Mexico
Fairchild*^k^*	GxL	G: 0.00 M: 0.00 L: 1.00	A	L	Coconut Grove, Florida, seedling of Collin Red, 1925
Fuerte*^k^*	GxM (M*^e^*)	G: 58.48 M: 41.52 L: 0.00	B		Atlixco, Mexico, budwood collected 1911
Ganter*^k^*	M	G: 0.00 M: 1.00 L: 0.00	B	M	Whittier, California, 1905
Gwen*^k^*	GxM*^c^* (G*^e^*)	G: 1.00 M: 0.00 L: 0.00	A		SCREC, Irvine, California, late 1960s
Hass	G (GxM*^b,c,e^*)	G: 1.00 M: 0.00 L: 0.00	A	G	La Habra Heights, California
Linda*^k^*	G	G: 1.00 M: 0.00 L: 0.00	B	G	Antigua, Guatemala, budwood collected 1914
Lyon*^k^*	G (GxM*^c^*) (M*^e^*)	G: 83.78 M: 16.22 L: 0.00	B	G	Hollywood, California, 1908
Mendez	GxM*^f^*	G: 1.00 M: 0.00 L: 0.00	A		La Habra Heights, California, 1926
Nabal*^k^*	G	G: 1.00 M: 0.00 L: 0.00	B	G	Antigua, Guatemala, budwood collected 1917
Nimlioh*^k^*	G	G: 1.00 M: 0.00 L: 0.00	B	G	Antigua, Guatemala, budwood collected 1917
*Ocotea botrantha^k^*	N/A	N/A	N/A	O	Unknown
Pequeno Charly	M*^b^*	G: 0.00 M: 1.00 L: 0.00	N/A	M	Unknown
*Persea donnell-smithii^k^*	N/A	N/A	N/A	O	Unknown
*Persea hintonii^k^*	N/A	N/A	N/A	O	Unknown
*Persea schiedeana* (CH-GU-01)	N/A	N/A	N/A		Mazatenango, Suchitepequez, Guatemala
Pinkerton*^k^*	GxM*^c^* (G*^e^*)	G: 81.00 M: 19.00 L: 0.00	A		Saticoy, California, late 1960s
Reed*^k^*	G*^c^*	G: 1.00 M: 0.00 L: 0.00	A	G	Carlsbad, California, putative Anaheim x Nabal progeny, 1960
Simmonds*^k^*	L	G: 0.00 M: 0.00 L: 1.00	A	L	Miami, Florida, seedling of Pollock, 1908
Taft*^k^*	G	G: 1.00 M: 0.00 L: 0.00	A	G	Seed from purchased fruit, likely Mexican origin, 1899
Thille*^k^*	GxM*^c^* (G*^e^*)	G: 1.00 M: 0.00 L: 0.00	B	G	Santa Paula, California, 1946
TopaTopa*^k^*	M	G: 0.00 M: 1.00 L: 0.00	A	M	Ojai, California, 1907
VC26*^k^*	L*^g^*	G: 0.00 M: 0.00 L: 1.00	A	L	Volcani Institute, Israel
Velvick	L*^h^*	G: 59.18 M: 0.00 L: 40.82	N/A		University of Queensland, Australia
Waldin*^k^*	L	G: 0.00 M: 0.00 L: 1.00	A	L	Homestead, Florida, 1909
Zutano*^k^*	M (GxM*^e^*)	G: 38.35 M: 61.65 L: 0.00	B		Fallbrook, California, 1926

G refers to Guatemalan, M to Mexican, and L to Lowland. The admixed percentage refers to our Admixture results with *K* = 3.

Rendón-Anaya *et al.* (2019).

University of California Avocado website (http://ucavo.ucr.edu/avocadovarieties/varietyframe.html).

California Avocado Society (1951).

Chen *et al.* (2009).

Illsley-Granich *et al.* (2011).

Ben-Ya’acov and Michelson (1995).

Anderson (2004–2005).

N/A – not available.

Denotes the designation for use in nonhybrid phylogenetic and population genetic analyses. L = Lowland (*n* = 5); M = Mexican (*n* = 3); G = Guatemalan (*n* = 10); W = wild (*n* = 3); O = Outgroup (*n* = 3). See text for details.

Collected from the UC ANR Research Station (Irvine, CA), with data generated for this study.

### Phylogeny and population structure

We performed PCA using VCFTools v0.1.17 git commit 954e607 (Danecek *et al.* 2011) and PLINK v1.9 (Purcell *et al.* 2007). All samples had < 50% missing data for *P. americana* and <75% for outgroup taxa. We created a maximum likelihood phylogeny with a reduced number of sites from SNPhylo (Lee *et al.* 2014) using IQ-TREE v1.6.12 (Nguyen *et al.* 2015) and the “PMB + F + G4” substitution model, as chosen by Model Finder (Kalyaanamoorthy *et al.* 2017). We employed the ultrafast bootstrap with 1,000 replicates to obtain support values. Admixture plots and analyses were performed with NGSadmix from the ANGSD package version 0.930 (build 2020 January 6, 13:30:06). CLUMPAK (Kopelman *et al.* 2015) was used to identify the best number across *K* = 1–10 groups, each with 10 replicates. To apply TreeMix (Pickrell and Pritchard 2012), we first filtered the SNP data containing 33 samples (all avocado accessions and 3 of the 4 outgroups because *P. schiedeana* proved to be a poor outgroup; see below) to keep sites without missing data. We then pruned the SNPs based on linkage disequilibrium using PLINK v1.90b6.16 (Purcell *et al.* 2007) with a 20 kb window, 5 kb step, and an r^2^ threshold of 0.1. The pruned SNPs in VCF format were then converted using the “vcf2treemix.sh” script included in the TreeMix package to create the input format. We ran TreeMix v1.13 with migration edges ranging from 1 to 6 with 5 replicates each. Finally, we used the Evanno method from the package OptM v0.1.6 (Fitak 2021) to determine the optimal number of edges.

### Population genetic analyses

To infer demographic histories, we used MSMC2 v2.1.1 (Mallick *et al.* 2016) with phased SNPs from the entire scaffolded assembly, which included the 12 pseudo-chromosomes and unplaced contigs. For each genetic group, we applied MSMC2 to the 3 accessions with the highest average sequencing coverage per genetic group (Guatemalan: Lyon, Carlsbad, Nimlioh; Lowland: Fairchild, Waldin, VC26; Mexican: Ganter, PequeñoCharly, TopaTopa). We created a mappability mask and a coverage mask (Supplementary Methods 1). Phase-informative SNPs (PIRs) were extracted for each sample as described in (Delaneau *et al.* 2013), which identifies reads that span at least 2 heterozygous SNPs; we then used the PIRs and the VCF files for each genetic group by chromosome as input to shapeit2 v2.r904 (Delaneau *et al.* 2013) for phasing. Finally, we used the “generate_multihetsep.py” script from the MSMC tools (https://github.com/stschiff/msmc-tools) to create MSMC2 input, taking into account mappability and coverage masks. To calculate the relative cross-coalescence rate (rCCR) across groups, we ran MSMC2 with all possible haplotype combinations between groups. To calculate split times, we used “plot_msmc.py” (Schiffels and Wang 2020) to estimate the time at rCCR = 0.5, based on a rate of 5.4e-9 mutations per site per year (Liang *et al.* 2019) and a generation time of 7 years.

To infer selective sweeps and to examine divergence between groups using *Fst*, we focused on a representative subset of accessions that (*1*) reduced nonrandom sampling in the Guatemalan group by removing 2 of the first- and second-degree relatives of Hass (Table 1), and (*2*) including only nonhybrid accessions, as inferred from Admixture analysis. For sweeps, we applied SweeD (Pavlidis *et al.* 2013) with default parameters on the scaffolded assembly, including unplaced contigs. To perform the analyses, we split the genome into nonoverlapping 10 kb windows created by bedtools v2.27.1 (Quinlan 2014), ignoring gaps, and focused on windows within the top 1% of the Composite Likelihood Ratio (CLR) statistic. For visualizing the results across chromosomes, we Loess smoothed across windows using the “geom_smooth” and “loess” methods with a span of 0.5 in ggplot (Wickham 2016), after assigning CLR values in gaps to zero. SweeD identifies the location of putative sweeps; to identify genes encompassed in that sweep, we included genes of 5 kb on either side of the location. Once genes were identified, we performed 2 types of analyses: GO enrichment, as described above, and the statistical significance of the number of shared sweep genes between groups. To evaluate significance, we permuted labels on genes (either sweep or nonsweep) within each race, recalculated the number of sweep genes in common between groups, and repeated the permutation 10,000 times. We used PLINK (Purcell *et al.* 2007) on 20 kb windows to calculate mean *Fst* for each window between samples, focusing on the top 1% windows.

## Results

### Gwen genome assembly and characterization

#### Gwen genome assembly

We generated 81.2 gigabases, equivalent to roughly 90 × coverage, based on the expected 1C genome size of 896 Mb (Arumuganathan and Earle 1991). We assembled PacBio SMRT reads using Canu (v 2.1), producing a genome of 1,456 Mb with 5,122 contigs, and then applied HapSolo (Solares *et al.* 2021) to remove putative secondary contigs (or haplotigs). The Canu + HapSolo (C + H) genome resulted in a primary assembly of 1,032 Mb, a longest contig of 17 Mb, a BUSCO score of 91%, and an N50 of 3.37 Mb (Supplementary Table 1). One useful measure of an assembly is the percentage of the assembly that is encompassed in the largest *x* contigs, where *x* represents the number of chromosomes (which is 12 for 1C *P. americana*). For the C + H assembly, this percentage was 17%—i.e. the 12 largest contigs represented 17% of the genome.

**Fig. 1.**
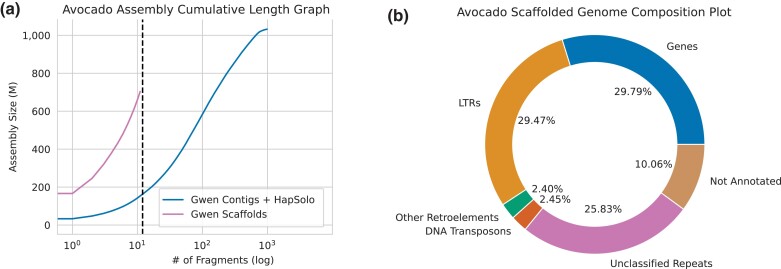
a) The cumulative sum assembly graph size (cdf) shows the size of the assemblies with the next largest consecutive contig (or scaffold) being added to the sum along the *x*-axis for the 2 Gwen assemblies (C + H and scaffolded). b) The annotation results for the Gwen C + H assembly, showing the percentage of the genome attributable to genes and different types of transposable elements, including DNA transposons, unclassified retroelements, and long terminal repeat (LTR) retroelements.

To improve contiguity, we anchored and scaffolded the C + H assembly using a published genetic map, based on a cross between Gwen and Fuerte (Ashworth *et al.* 2019). This exercise resulted in 12 scaffolds that were assigned to 12 linkage groups, along with unplaced contigs. Scaffold N50 improved ∼18-fold (to 61.9 Mb) over the 3.37 Mb contig N50. The 12 largest scaffolds (pseudo-chromosomes) represented 78% of the expected genome size of 896 Mb (Fig. 1a), which is superior to some previously published Hass genomes (Supplementary Table 1). Although each chromosome could be identified among the 12 largest scaffolds, we interpret the smaller-than-expected genome size to imply that the density of the genetic map limited resolution. The first Hass primary assembly also decreased substantially in size when it was anchored to a genetic map (Rendón-Anaya *et al.* 2019)—e.g. only 47% of the Hass assembly could be anchored, resulting in a scaffolded genome of 421.7Mb, while the second lacked any scaffolding (Sharma *et al.* 2021).

#### Genome annotation

We annotated the C + H assembly and scaffolded assemblies by first identifying the repetitive content (see Methods). We estimated that ∼61% of the Gwen genome consisted of repetitive elements (Fig. 1b), of which 52% were long terminal repeat (LTR) retroelements (with more than half of these being *Gypsy* elements) and another 40% were unknown repeats (Fig. 1b). We masked repeats before annotating genes (see Methods), inferring 36,993 genes on the 12 pseudo-chromosomes and 12,457 in unplaced contigs. We compared these numbers to the Hass genome annotation (Rendón-Anaya *et al.* 2019), which reported 33,378 genes. We filtered this set to remove potential duplicates, yielding 25,211 genes. Of the 25,211, 94% were present in our scaffolded assembly; thus, our annotation corroborates most previous genic inferences but also annotates ∼1.5-fold more genes.

#### Genic hemizygosity

Recent studies have suggested that diploid genomes may be replete with genic hemizygosity. We assessed genic hemizygosity by remapping raw PacBio reads to the 12 pseudo-chromosomes of the scaffolded assembly, by inferring structural variants, and then by filtering for variants that overlapped annotated genes (see Methods). With this approach, we estimated that 3.8% of genes were hemizygous in the Gwen genome. This estimate serves as a benchmark that can be used to compare other diploid systems (see Discussion).

### Analysis of *P. americana* genetic structure

#### Classification of groups and hybrid accessions

Given the Gwen reference genome, we performed a preliminary study of genetic variation and evolutionary genomics across avocados. To do so, we amassed a whole-genome resequencing data set of 30 *P. americana* accessions and 4 putative outgroups, all with at least 14 × coverage (Table 1). The sample represented the 3 botanical groups of avocado, based on historical designations, historically important cultivars (Chen *et al.* 2009), and both A and B flowering types (Table 1). Given the resequencing data, we mapped reads to the scaffolded assembly (including unplaced contigs) and identified 23,938,386 biallelic SNPs within our entire *P. americana* sample and 32,235,263 biallelic SNPs when the 4 outgroups were analyzed with the *P. americana* samples.

**Fig. 2.**
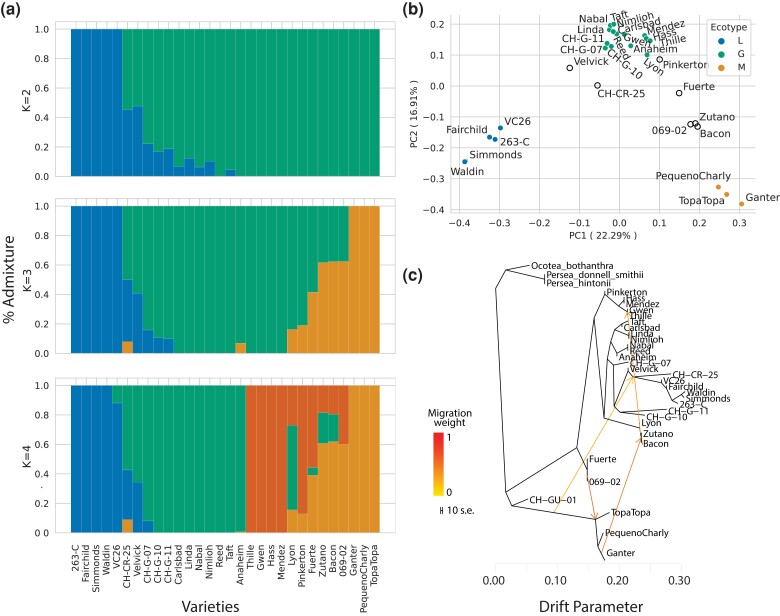
a) Admixture plots generated from the entire sample of 30 avocado accessions, using SNPs based on the Gwen scaffolded assembly. The plots show the inferred groups with *K* = 2, *K* = 3, and *K* = 4, which was inferred to be the most likely number of clusters with this data set. The *K* = 4 groups include the 3 historical groups (Mexican in orange on far right, Guatemalan in green in the center, and Lowland in blue on the left) and another group in red with accessions closely related to Hass. b) A PCA analysis of SNP diversity. Each dot represents an individual, and the color of the dot represents accessions with >80% assignment to a single group, including Guatemalan (G), Mexican (M), and Lowland (L). Open dots represent likely hybrid accessions (c) Results of TreeMix, with the optimal 4 edges. The accessions, including 3 outgroups are labeled.

SNPs were then subjected to 3 types of clustering analysis—admixture mapping, principal component analysis (PCA), and phylogenetic inference—to define groups of accessions based on whole-genome data. We first investigated relationships among accessions using admixture mapping. The most highly supported analysis contained *k* = 4 groups: the 3 previously recognized botanical groups (Lowland, Mexican, Guatemalan) and a series of cultivars related to Gwen and Hass (Fig. 2a). This last group included Mendez, a somatic mutation of Hass (Illsley-Granich *et al.* 2011; Schaffer *et al.* 2013), and other close relatives like Thille. We suspect that this last group was defined by over-sampling accessions with first- or second-degree relationships to Hass. Indeed, the removal of 2 accessions from the “Hass group” yielded *k* = 3 as the most supported number of groups, with the 3 groups representing the previously recognized groups (Supplementary Fig. 1). Given this result and the historical designation of *k* = 3, we reported the proportion of each accession based on *k* = 3 groups (Table 1). These admixture maps reflect a hybrid history of some accessions, including Velvick, Fuerte, Bacon, Zutano, and also an accession (CH-CR-25) that was thought to represent a new racial ecotype—var. *costaricensis* (Ben-Ya’acov *et al.* 2003; Rendón-Anaya *et al.* 2019).

Somewhat surprisingly, the admixture analyses also indicated that Hass is 100% Guatemalan, whereas previous work defined it as >50% Mexican (Rendón-Anaya *et al.* 2019) or 58% Guatemalan (Chen *et al*., 2009). We note that our results are consistent across Hass and its close relatives like Gwen, which is a grandchild of Hass, and Mendez. We also remark, however, that the reduced dataset suggested that Hass has a small genetic proportion (< 25%) attributable to the Mexican group (Supplementary Fig. 1), which is still far less than previous studies. Overall, these results suggest that Hass originated primarily from the *guatemalensis* group, with the complete dataset identifying a solely Guatemalan origin.

Second, we applied PCA to the *P. americana* data without outgroups. The results verified many of the admixture analyses, including clusters that represented previously identified Mexican, Guatemalan, and Lowland groups (Fig. 2a). The PCA consistently placed Hass and its relatives squarely within the Guatemalan group, but also clearly indicated that some accessions fell between groups on the PCA (e.g. Velvick), reinforcing hybrid origins. Furthermore, economically important accessions like Bacon and Zutano have been inconsistently inferred or assigned as hybrids (Chen *et al.* 2009), but our whole-genome analyses suggest likely hybrid origins (Fig. 2, a and b and Table 1). For completeness, we also applied PCA to a data set with the 4 putative outgroup taxa. The genetic placement of 3 species (*P. donnell-smithii* Mez*, P. hintonii* C.K. Allen*, O. botrantha* Rohwer) was consistent with their expected outgroup status, but *P. schiedeana* Nees clustered near the avocado ingroup (Supplementary Fig. 1). The *P. schiedeana* results are consistent with previous suggestions that it hybridizes with avocado (Ashworth and Clegg 2003), indicating it should not be used as an outgroup for evolutionary analyses as it has previously (Rendon-Ayala *et al*., 2019).

Finally, we investigated relationships among accessions and the 3 legitimate outgroups using 2 phylogeny-based methods. The first was TreeMix, which can reveal the potential presence of historical gene flow. Given that populations were difficult to define in our sample, due to apparently recent hybrid origins, we applied TreeMix to individuals and identified an optimum of 4 edge migration events (Fig. 2c). Unsurprisingly, TreeMix inferred migration events that likely reflect recent breeding history—e.g. directional migration between the pure Mexican accessions (represented by Topa Topa, PequenoCharly, and Ganter in Fig. 2c) and hybrid accessions like Zutano and Bacon. TreeMix also suggested ancient migration between the lineage leading to the Mexican group toward the base of the lineage representing the Costa Rican sample (CH-CR-25). This observation lends credence to the idea that var. *costaricensis* represents a distinct ecotype (Ben-Ya’acov *et al.* 2003), perhaps born of an ancient hybridization event.

**Fig. 3.**
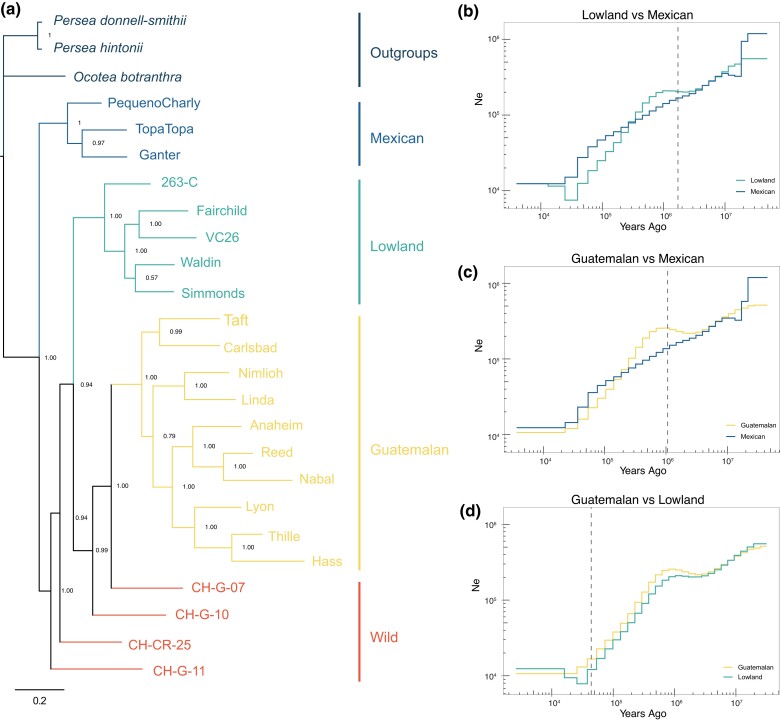
a) Genome-wide phylogeny of *P. americana* ingroup with 3 species outgroups. Accessions were chosen if they had 80% of higher assignments to a race, including putatively wild accessions (CH-G-07, CH-G-10, CH-G-11, and CH-CR-25). The numbers on the nodes represent bootstrap proportions. b) Demographic inference based on the Mexican and Lowland samples. The x-axis represents the time from present to past, and the y-axis is inferred as effective population size (Ne). The vertical dashed lines represented the estimated divergence time between the 2 groups. c) Like b, except for Mexican and Guatemalan samples. d) Like b, except for the Lowland and Guatemalan samples.

We also created a consensus phylogenetic tree to investigate relationships among the 3 historical groups. Because we intended to investigate phylogenetic history (and not the history of recent hybrid events due to breeding), we based this tree on a representative set of nonhybrid accessions that had high support (*Qi* > 80%) for inclusion in a single genetic group at *K* = 3 (Vigouroux *et al.* 2008; Castillo *et al.* 2010), reduced sampling of the near-relatives of Hass, and on a reduced number of SNPs to limit linkage disequilibrium (see Methods). The phylogeny had median bootstrap support of 88.5% for all nodes and strong support (> 76%) for nodes that separated the botanical groups (Fig. 3a; Table 1). Moreover, the accessions from each group formed well-supported monophyletic clades, justifying treating each named group as historically separate. An interesting feature of the rooted phylogeny is that the Mexican group is an early-diverging sister group, suggesting an early split from the Lowland and Guatemalan lineages and a separate domestication of the Mexican avocado. Another interesting feature is the placement of putatively wild accessions, CH-G-07 and CH-G-10. These accessions, which were from the study of Rendon-Ayala *et al*. (2019) and designated as wild in their analyses (Table 1), intercalate the Lowland and Guatemalan clades. Assuming these accessions are, in fact, wild, as opposed to feral escapees, their placement on the tree reinforces the idea that the Lowland and Guatemalan groups were domesticated separately (Furnier *et al.* 1990; Ashworth and Clegg 2003; Rendón-Anaya *et al.* 2019).

### Population diversity analyses

Our sampling was designed to include representative samples from each botanical group. However, analyses of the genetic structure identified hybrids that were unlikely to be helpful for inferring the historical dynamics of specific groups. Accordingly, we focused population genetic analyses on representative samples with *Qi* > 80% (Fig. 3a). This resulted in different sample sizes for the 3 groups, with the Mexican sample being the smallest at *n* = 6 chromosomes from 3 diploid individuals (Table 1). These sample sizes likely limit inferential power for some analyses, but we nonetheless retained > 10 million SNPs within each sample, with nucleotide diversity (π) similar among groups, at ∼0.0035 per nucleotide site (Supplementary Table 2).

#### Demographic history

We applied the Sequentially Markovian Coalescent to infer 2 distinct aspects of the history of botanical groups. The first was to infer whether any experienced a domestication bottleneck or other dramatic demographic event, recognizing that perennial crops often lack a signature of such events (Gaut *et al.* 2018). After applying MSMC to the 3 individuals with the highest coverage from each group (to ensure equal sample sizes across groups; see Methods), the results indicated a consistent reduction of *Ne* over time but without any evidence of a particularly notable bottleneck or rapid post-bottleneck expansion (Fig. 3b). The second was to estimate the timing of the split of botanical groups; the divergence times complemented the phylogeny by indicating an early split (∼1.3 million years ago) of the Mexican group. In contrast, the Lowland and Guatemalan groups diverged more recently, at ∼44,000 years ago. These split times predate expected domestication times, and thus, likely reflect divergence times between ancestral wild progenitor populations (Fig. 3b).

#### Selective sweep mapping

For the 3 sets of samples representing potentially distinct domestication events, we investigated 2 additional features of their evolutionary genomics. The first was sweep mapping within each botanical group and the second was the divergence between groups, as measured by *Fst*. We performed these analyses to assess whether similar (or entirely different) sets of genes bear marks of selection across groups, to investigate whether highly differentiated chromosomal regions between groups overlapped with selected genes, and to generate a list of candidate-selected genes with potential functions.

**Fig. 4.**
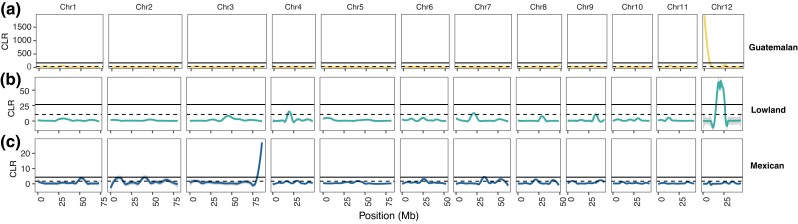
Loess smoothed composite likelihood values reflecting potential regions in samples from each of the 3 botanical groups: a) Mexican, b) Lowland, and (c) Guatemalan. Each panel represents the 12 scaffolded chromosomes, with chromosome number and location (in Mb) provided above and below each individual graph. In each graph, the dashed lines represent 1% and 5% cutoff values for significance; 95% Confidence Intervals are indicated by gray shades. Note the different scales of x-axes among the groups.

Sweep mapping relied on the CLR statistic and focused on 10 kb nonoverlapping genomic windows of the scaffolded assembly. Using an empirical cutoff of 1%, we identified 1,300 windows from each race, for which 638, 436, and 92 had genes in the Mexican, Lowland, and Guatemalan samples, respectively (Supplementary Table 3). Given these genes, we first hypothesized that separate domestication events may have targeted particularly important sets of genes in parallel. Visually, based on smoothed curves of the CLR statistic, this did not appear to be the case (Fig. 4), because putative sweep regions differed markedly among samples, although both the Lowland and Guatemalan samples had prominent sweep regions on chromosome 12. However, we also asked the question more formally by calculating the number of shared sweep genes between pairs of groups and then permuting labels (CLR, non-CLR) to test significance. We found no enrichment for shared CLR genes between the Guatemalan sample, and either the Mexican (*P* = 0.3692; 2 shared genes; Supplementary Table 4) or the Lowland samples (*P* = 1.00; 0 shared genes). However, the Lowland and Mexican groups had 18 CLR genes in common, a number significantly higher than random expectation (*P* < 0.0001) (Supplementary Table 5).

We evaluated sets of selected genes using GO enrichment. In the Guatemalan race, for example, the putative swept genes were enriched for functions related to fruit ripening, fruit development, anatomical structure maturation, and other functions (Supplementary Fig. 3). Since these functional enrichments reflect properties potentially associated with domestication, we consider this genic set to be noteworthy. In contrast, we did not identify the enrichment of gene function related to fruit maturation or suspected domestication traits for the Mexican and Lowland samples (Supplementary Figs. 4 and 5), perhaps reflecting statistical uncertainty due to their small sample sizes. We did, however, identify gene enrichments related to functions s stress response, terpene production, and metabolic processes. The set of shared 18 genes was also particularly interesting, due to the potential for parallel selection during separate domestication events. A subset of the genes had positive hits to functional annotation databases (Supplementary Table 5). However, with the possible exception of 2 genes that function in sugar transport, none have functions related to obvious domestication traits like fruit size or development.

#### Divergence mapping

We next examined divergence between groups using *Fst* based on 20 kb nonoverlapping windows along scaffolded chromosomes, focusing on regions that include the top 1% of *Fst* scores (Supplementary Fig. 6). For example, for the pairwise comparison between the Mexican and Lowland samples, we identified peaks containing 396 genes, with similar numbers for the other pairwise comparisons (Mexican–Guatemalan 387 genes; Lowland–Guatemalan 384 genes) (Supplementary Table 6). In contrast to the potential for parallel domestication pressures on some genes, we expected *Fst* results to be enriched for genes that contribute to agronomic differences between the 2 groups. Several genes in the top 1% were related to disease resistance and response, particularly drought and cold/heat response. We performed GO enrichment on these samples of genes (Supplementary Figs. 7–9), finding enrichment for light stimulus, pollen recognition, and several cellular and metabolic processes. We also assessed whether genes were shared between *Fst* and sweep analyses, hypothesizing that the selection of genes within 1 race could contribute to genetic divergence between groups. To perform this analysis, we identified genes shared between *Fst* and CLR analyses—e.g. we compared the set of 396 genes from Mexican–Lowland *Fst* analysis to the 436 CLR genes in the Lowland sample. We found 10 genes shared between the 2 lists, which was an enrichment relative to random expectation (permutation *P* < 0.0028). The number of shared genes between *Fst* and CLR analyses was higher than expected for 4 of 6 comparisons (Supplementary Table 4). The shared genes again constitute another set of credible domestication or improvement genes (Supplementary Table 7).

### Contrasting A and B flowering types

To date, the genetic causes of heterodichogamy have not been identified in any system (Endress 2020). For that reason, we thought it worthwhile to explore genetic factors associated with type A and B flowers. Many of our resequenced accessions had known flowering types, with samples of *n* = 13 A types and *n* = 9 B types in total (Table 1). Importantly, within each flowering type, the samples traversed genetic groups—e.g. the A types included samples from each of the 3 groups (Mexican, Guatemalan, and Lowland), and the B types were distributed across the Mexican, Guatemalan and hybrid samples. Given the distribution of A and B types across groups, we thought that contrasting the 2 samples may provide preliminary insights into genomic regions that contribute to this interesting phenotype, perhaps without being overly confounded by population structure.

**Fig. 5.**
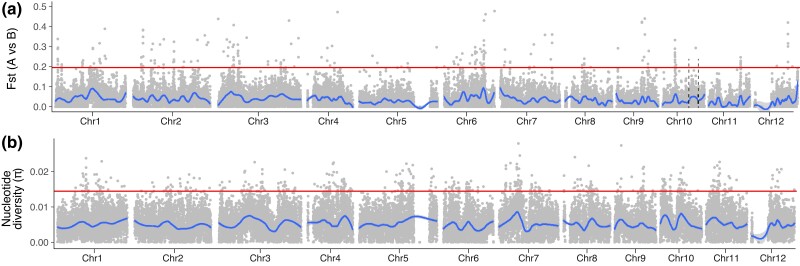
a) A genomic plot of *Fst* windows contrasting the A and B flowering type accessions. The horizontal line denotes the top 1% *Fst* peaks, which include 466 genes. The black dashed lines on chromosome 10 show the approximate location of a previously identified QTL for flowering type b) A plot of nucleotide diversity (π) across the 12 pseudo-chromosomes based on the “pure” Guatemalan (*n* = 20 chromosomes) sample. The horizontal lines indicate an empirical 1% cutoff.

Therefore, we performed *Fst* analyses between the 2 groups, producing a plot with peaks of differentiation between types (Fig. 5a). The average value of *Fst* was low (at 0.038) compared to *Fst* differentiation between groups (average *Fst*: Guatemalan vs Mexican = 0.241, Guatemalan vs Lowland = 0.223; Lowland vs Mexican = 0.325) (Supplementary Fig. 6), reflecting again the fact that the A vs B samples do not represent highly differentiated samples. There were, nonetheless, regions of visually compelling *Fst* peaks between flowering types—e.g. evident peaks on chromosomes 6 and 10, among others. These peaks could be an artifact of the population histories of the samples, but they may also contain genes that differentiate the A and B morph. Consistent with the latter interpretation, the set of 466 genes within the top 1% of *Fst* windows (Supplementary Table 8) were enriched for functions—like pollination, floral development, and photoperiodism—that likely contribute to heterodichogamy (Supplementary Fig. 10). Given the functional enrichments, we explored the list of 466 genes to find genes related to floral development and timing, yielding several genes with homologs that affect floral development, circadian rhythm, photoperiodism, and the production of volatiles (Table 2). Reasoning that these results should be consistent for the subset of A vs B accessions within the Guatemalan group, we repeated the analyses in the Guatemalan sample, finding again an *Fst* signal for 4 of the candidates (Table 2). Finally, our results identified a prominent peak on Chromosome 10, but not in a location that overlapped with a previously identified QTL region for the flowering type (Chen *et al.* 2009) (Fig. 5a).

**Table 2.** Candidate genes found in *Fst* peaks between the samples of A- and B-type flowering accessions with apparent functions in flower development.

Gene*^a^*	Homolog*^b^*	Homolog function
ChrU.ver1.g447950, ChrU.ver1.g387390	*VOZ1* ^c^ (*Arabidopsis thaliana*)	Regulates *Flowering Locus C (FLC)* and *Flowering Locus T (FT)*
ChrU.ver1.g414850	*CCR1* (*Petunia hybrida*)	Biosynthesis of volatile compounds in flowers
Chr5.ver1.g224860, ChrU.ver1.g377230	*EOBI* (*Petunia hybrida*)	Transcription factor for volatile compounds in flowers
Chr10.ver1.g45990	SPA1 (*Arabidopsis thaliana*)	Controls normal photoperiodic flowering and regulates circadian rhythms
ChrU.ver1.g440620, ChrU.ver1.g440630	KCS11*^c^* (*Arabidopsis thaliana)*	Response to cold and light stimulus
Chr3.ver1.g159200	AP2 (*Arabidospis thalian*a)	Transcriptional activator that promotes early floral meristem identity
Chr5.ver1.g230020, Chr5.ver1.g230030	MTERF2*^c^* (*Arabidopsis thaliana*)	Transcription termination factor; knock-outs delay growth and flowering
Chr1.ver1.g24210	NPR5*^c^* (*Arabidopsis thaliana*)	Acts redundantly with *BOP2*. *BOP1/2* promote leaf and floral meristem fate and determinacy in a pathway targeting *AP1* and *AGL24*
ChrU.ver1.g393700	CSU2 (*Arabidopsis thaliana*)	Inhibits *COP1*, which is involved in seedling growth and photoperiodism in flowering

Gene number in Gwen annotation file.

A homolog to the gene, as identified by functional analyses with SwissProt, with the species in which the homolog was identified. [Note: *SPA1* is not present in the scaffolded assembly and was identified on *Fst* analyses of the C + H assembly.]

Also identified in *Fst* analyses based on the A vs B sample of only Guatemalan accessions.

We sought another piece of evidence to provide additional support for any of the genes in Table 2. We first wondered whether heterodichogamy could be caused by dosage effects and, therefore, investigated whether any of the 466 *Fst*-based genes were hemizygous in Gwen, an A-type flower morph. Of the 466 genes, 17 were hemizygous, a percentage (3.6%) nearly identical to the genome average (3.8%), suggesting hemizygosity was not a refining criterion. None of the hemizygous genes were related to floral function (Table 2). Second, we hypothesized that the A vs B polymorphism has been subject to balancing selection, given that heterodichogamy predates the diversification of *P. americana* (Renner 2001). If true, we expected causal genomic region(s) to have especially high levels of nucleotide diversity within a sample that contained both flowering types. We examined the Guatemalan sample to scan for 5 kb regions of high nucleotide diversity. While there were some weakly apparent peaks of diversity, their locations did not correspond with *Fst* peaks between the A and B flowering types (Fig. 5b); none of the genes in Table 2 were among the set of 1% most diverse genes. Overall, 3 genes were found in both *Fst* peaks and high diversity windows—a Leucine-Rich Repeat gene (Geneid Chr6.ver1.g275850) and 2 genes similar to the mitochondrial transcription termination factor *MTERF2* (Geneids Chr5.ver1.g230020 and Chr5.ver1.g230030).

## Discussion

We have assembled and annotated the genome of Gwen avocado. Annotation identified ∼65% of the genome as repetitive with 49,450 genes (Fig. 1b). The latter number is almost 2-fold higher than those predicted on the Hass genome (Rendón-Anaya *et al.* 2019), but approaches the 63,000 genes predicted in a transcriptome analysis (Chabikwa *et al.* 2020). Among the genes, 3.8% have been detected as hemizygous, due to structural variants that affect >20% or more of the coding region. This genic hemizygosity value is intermediate among a range that includes perennial grapevines (>12%) (Vondras *et al.* 2019; Zhou *et al.* 2019), an outcrossing annual relative of rice (8.9%) and selfing rice (<<1%) (Kou *et al.* 2020). Given that avocado is clonally propagated, we naively expected hemizygosity to be similar to the grapevine. The low value for Gwen probably reflects a low divergence between its parents and, perhaps, the fact that the sampled Gwen tree does not yet have an extensive history of clonality, which promotes the maintenance of hemizygous deleterious variants (Zhou *et al.,* 2017). We do know that genic hemizygosity contributes to trait differences among grapevines (Carbonell-Bejerano *et al.* 2017); it will be interesting to monitor similar issues in avocados for accessions like Mendez, a somatic variant of Hass.

We generated the Gwen genome as a tool for breeding but used it here for investigating questions about the evolutionary history of *P. americana*—e.g. as a reference to address questions about the number of domestication events and the timing of divergence among botanical groups. Our analyses are consistent with 3 independent domestication events (Furnier *et al.* 1990; Ashworth and Clegg 2003; Rendón-Anaya *et al.* 2019), because admixture, PCA, and phylogenetic analyses clearly differentiated among the 3 botanical groups (Figs. 2 and 3). We also estimated divergence times (Fig. 3), which varied from ∼40,000 years between the Lowland and Guatemalan groups to >1.0 My between the Mexican and the 2 other groups. These estimates are much older than the expected domestication times for perennial crops (Miller and Gross 2011; Gaut *et al.* 2015), and hence, likely reflect divergence among wild lineages that eventually became the sources for domestication. The early divergence among groups may have been driven in part by ecological differences among regions, especially given the evidence that wild germplasm in Mexico is genetically subdivided by elevation (Chen *et al.* 2009).

Our analyses have also provided insights into the inter-racial hybrid origins of some cultivars, representing the first whole-genome insights for most of our samples. Many of our results confirmed findings based on microsatellites and other marker types (Ashworth and Clegg 2003; Chen *et al.* 2008, 2009)—e.g. accessions such as Zutano and Bacon were previously thought to be hybrids between botanical groups, which was confirmed in our analyses (Table 1)—but also offered surprises. The most notable was the genetic history of Hass, which was traditionally thought to be of Guatemalan origin (Ashworth and Clegg 2003), but had been inferred to be roughly 50% Guatemalan and 50% Mexican from genetic analyses (Chen *et al.* 2009; Rendón-Anaya *et al.* 2019). We find, however, that Hass falls predominantly into the Guatemalan group, as do the close relatives of Hass in our sample (Supplementary Fig. 2). Although we do not know the source of disagreement among studies, the genetic provenance of Hass—the most cultivated accession in the world—may not yet be fully resolved.

We removed hybrid accessions to define “pure” samples for population genetic analyses and to investigate the history of groups. The resulting sizes of samples varied and likely contributed to some of the variances in results across groups. For example, the smaller sample size of the Mexican sample likely led to false positives for sweep genes (*n* = 6 chromosomes with 638 CLR genes) compared to the Guatemalan sample (*n* = 20 chromosomes with 92 CLR genes). We have, nonetheless, found a few compelling patterns. For example, the set of high-CLR genes in the Guatemalan race was enriched for functions related to fruit development, likely a trait targeted for domestication. This gene set is, then, fitting for further study and has the potential to help disentangle the origin of some agronomic traits. In addition, some genes were shared as putative domestication genes more often than expected, including 18 genes between the Lowland and Mexican groups. In theory, these genes could represent genomic regions that are particularly prone to maintaining the history of sweeps (i.e. low recombination regions), but genome-wide patterns of the CLR statistic do not superficially suggest this as the cause (Fig. 4). We consider it more likely that these genes represent parallel selection pressures for independent domestication events, but we have few insights into how they may have contributed to domestication traits.

We used similar approaches to investigate regions of genomic divergence between samples defined either by race or flowering type. The latter yielded the most promising insights, representing (to our knowledge) the first attempt to define genomic regions that may contribute to heterodichogamy. Our inferences are at best preliminary, but they have yielded some interesting candidates, including homologs to *VOZ1*, which regulates *FLC*, a transcription factor that functions as a repressor of floral transition and contributes to temperature compensation of the circadian clock (Mitsuda *et al.* 2004; Yasui *et al.* 2012); *SPA1*, which contributes to the regulation of circadian rhythms of flowering processes in *A. thaliana* (Ishikawa *et al.* 2006); and *APETALA2*, which binds to thousands of loci in the developing flower and controls various aspects of floral development and organ identity (Yant *et al.* 2010). We have also documented a prominent peak on chromosome 10 (Fig. 5a), the same chromosome that housed a flowering type QTL (Ashworth *et al.* 2019). However, our peak does not overlap with the QTL region identified previously, and we also found no particularly noteworthy candidate genes under the peak identified in this study. It is unclear why the 2 studies do not correspond, but potential reasons include a low resolution for the QTL study, potential structural variants between the parents of the QTL study or between Gwen haplotypes, and potential nonincorporation of key contigs into the scaffolded assembly.

Interestingly, none of our candidate genes for heterodichogamy bear an obvious signal of balancing selection, which is something we hypothesize should be present for a long-lived genetic polymorphism that contributes to A vs B flowering types, perhaps similar to the S-locus of *Arabidopsis* species (Roux *et al.* 2013). The few genes that do overlap between diversity and divergence seem unlikely to affect heterodichogamy [although it should be noted that *MTERF2* knock-outs affect flowering and plant growth (Lee *et al.* 2021)]. There are several reasons why it might be difficult to detect a balancing polymorphism if there is indeed a balanced polymorphism, but these candidate genes, nonetheless, suggest a way forward to study this interesting biological phenomenon. Future work will focus on additional genome sequencing and analyses of A vs B genomes, on characterizing segregation patterns of polymorphisms within candidate genes in a larger sample of A vs B avocado accessions, and also on searching for trans-specific polymorphisms (Charlesworth 2006) across species of the Lauraceae, since heterodichogamy is also found in other Lauraceae species (Renner 2001).

## Supplementary Material

jkac323_Supplementary_Data

## Data Availability

The data for this study were submitted to NCBI under BioProject: PRJNA758103, which contains all raw PacBio and Illumina sequencing data, as well as the scaffolded genome assembly. A gff file describing the genes and the scaffold annotation files are available at Zenodo: https://doi.org/10.5281/zenodo.6392169. Published resequencing data used in this study from Rendon-Ayala *et al*. (2019) were from NCBI numbers: SRR8295599, SRR8295600, SRR8295601, SRR8295602, SRR8295603, SRR8295604, SRR8295605, SRR8295607, SRR8295608, SRR8295609, SRR8295610, and SRR8295611. The RNAseq data used for gene annotation were downloaded from NCBI with accession numbers SRR6116327, SRR6116328, SRR6116329, SRR6116330, and SRR2000042. The scripts used for analyses are available from https://github.com/GautLab/avo_ref_paper Supplemental material is available at G3 online.

## Notes

*Communicating editor: M. Hufford*
