# Supplementary material for: Insights into the domestication of avocado and potential genetic contributors to heterodichogamy
Source: G3 (Bethesda). 2022 Dec 8;13(2):jkac323. doi: 10.1093/g3journal/jkac323 (PMC9911064; doi:10.1093/g3journal/jkac323)
Supplement: jkac323_Supplementary_Data [file jkac323_supplementary_data.pdf]

_G3_ Supporting Information

Insights into the domestication of avocado and potential genetic contributors to heterodichogamy

Edwin Solares*, Abraham Morales-Cruz*, Rosa Figueroa Balderas, Eric Focht, Vanessa E. T. M. Ashworth, Andrea Minio, Dario Cantu, Mary Lu Arpaia and Brandon S. Gaut

The following Supporting Information is available:

Fig. S1 Additional admixture plots based on a subset of *P. americana* accessions.
Fig. S2 Additional PCAs with outgroups.
Fig. S3 The outcome of GO enrichment analysis for the pure Guatemalan sample (n =10), based on the set of 92 candidate genes (Table S4) detected by selective sweep mapping.
Fig. S4 The outcome of GO enrichment analysis for the pure Lowland sample (n =5), based on the set of 436 candidate genes (Table S4) detected by selective sweep mapping.
Fig. S5 The outcome of GO enrichment analysis for the pure Mexican sample (n =3), based on the set of 683 candidate genes (Table S4) detected by selective sweep mapping.
Fig. S6 Plots illustrating *Fst* across the 12 scaffolded pseudo-chromosomes.
Fig. S7 The outcome of GO enrichment analysis based on the set of 401 candidate genes (Table S7) detected by *Fst* divergence analysis between the Mexican and Lowland pure sample.
Fig. S8 The outcome of GO enrichment analysis based on the set of 394 candidate genes (Table S7) detected by *Fst* divergence analysis between the Mexican and Guatemalan pure samples.
Fig. S9 The outcome of GO enrichment analysis based on the set of 385 candidate genes (Table S7) detected by *Fst* divergence analysis between the Lowland and Guatemalan pure samples.
Fig. S10 The outcome of GO enrichment analysis based on the set of 466 genes (**Table S9**) detected by *Fst* divergence analysis between the A and B Flowering Types.

Table S1 Assembly Metrics compared across avocado genome assemblies
Table S2 Number of biallelic SNPs and nucleotide diversity per base pair (p) within the pure samples of botanical races.
Table S3 List of genes under CLR peaks in each of the three samples representing botanical races. (see Supplementary Excel File)
Table S4 The outcome of enrichment analyses for comparing genes either inferred to be under selection between two races by Fst and sweep mapping
Table S5 Lists of 20 genes under CLR peaks that are shared between races with functional annotation information. (see Supplementary Excel File)
Table S6 List of genes under Fst peaks in pairwise comparisons (see Supplementary Excel File)
Table S7 List of the 45 genes that were identified as being under both 1% *Fst* peaks and under 1% CLR peaks, with functional annotation information.
Table S8 List of 466 genes that were under Fst peaks when comparing A flowering types to B flowering types and that were the basis for GO enrichment analysis. (see Supplementary Excel File)

Methods S1 A brief description of generating mapping and coverage masks for demographic analyses.

Fig. S1 Admixture plots based on removing two close relatives of Hass: Mendez (a purported somatic mutation of Hass), Gwen (a grandchild of Hass). With these samples, the optimal grouping is K=3 (middle graph), corresponding to Guatemalan, Mexican, Lowland and Hass groups.

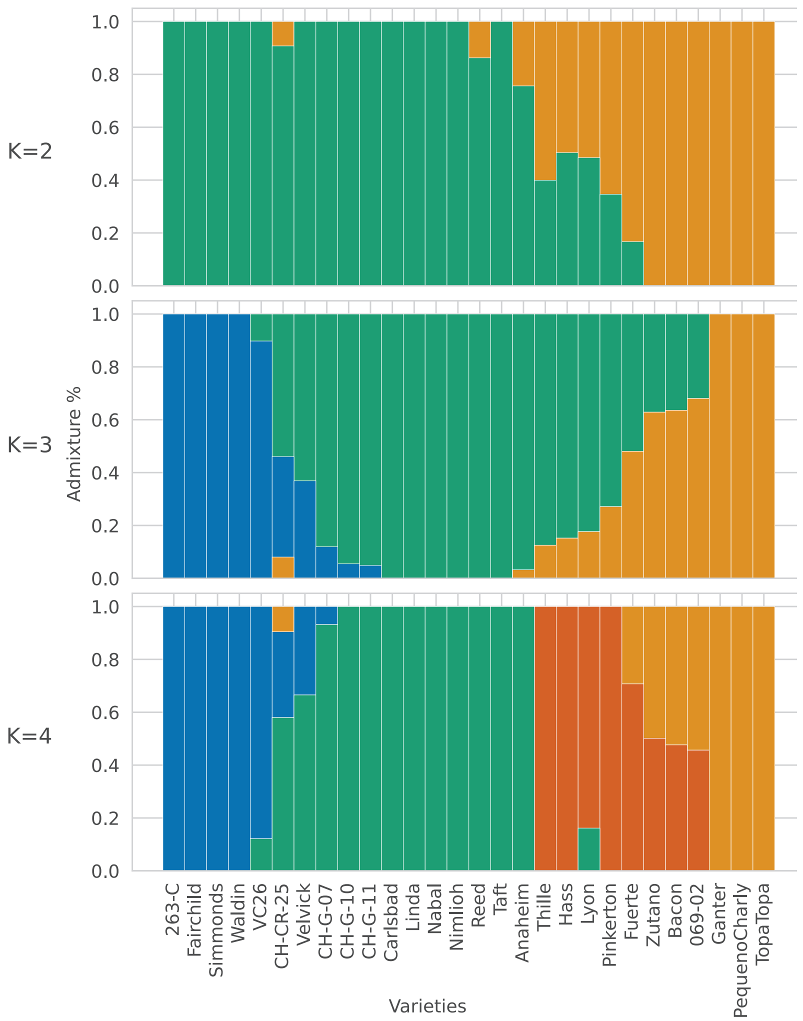

Fig. S2 Additional PCAs based on SNPs. **Top:** Avocado samples including *P. scheideanna* (CH-GU-01), showing it groups within the avocado samples. **Bottom:** The PCA includes all samples used in our analysis, including outgroups. Located in the far left are the *Persea* outgroups and in the top is the *Octea* outgroup, with *P. scheideanna* (CH-GU-01) also shown. The keys for each figures provides a rough estimate of grouping within avocados: M = Mexican, L = Lowland, G=Guatemalan, CR = Costa rican, Psch = *P. scheideanna,* hybrids (GxM, LxG), with N/A as outgroups.

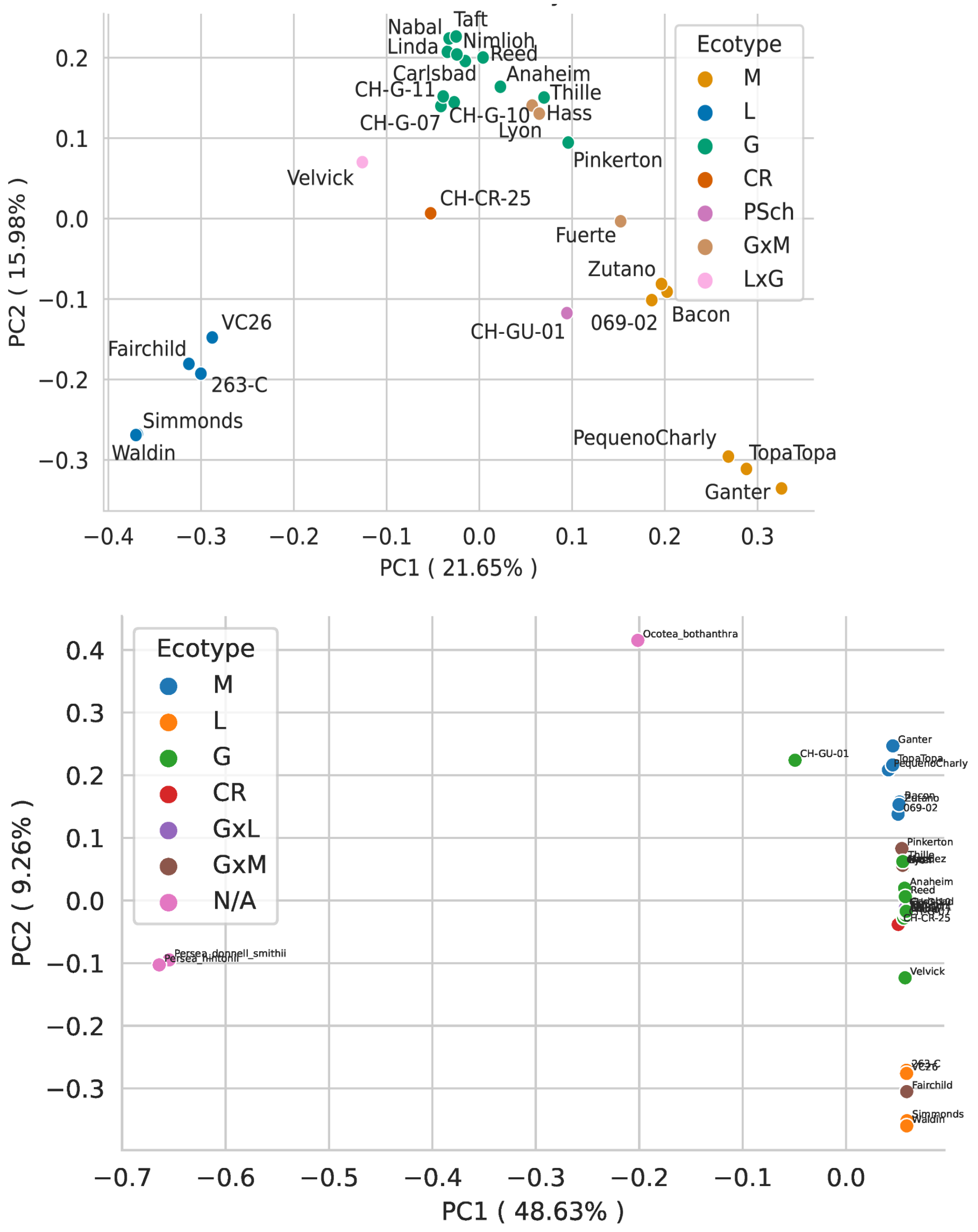

Fig. S3 The outcome of GO enrichment analysis for the pure Guatemalan sample (n =10), based on the set of 92 candidate genes (Table S4) detected by selective sweep mapping with SweeD. Both graphs were generated by blast2GO, and both include only significant categories as measured by a p-value of $p < 0.05$ by a Fisher's Exact Test, as corrected for multiple tests by the blast2GO program. The graphs differ in the specificity; the top graph was generated to include general terms, while the lower graph used the "reduce to most specific option" to report terms at the most specific level in the gene ontology enrichment directed acyclic graph (DAG) file.

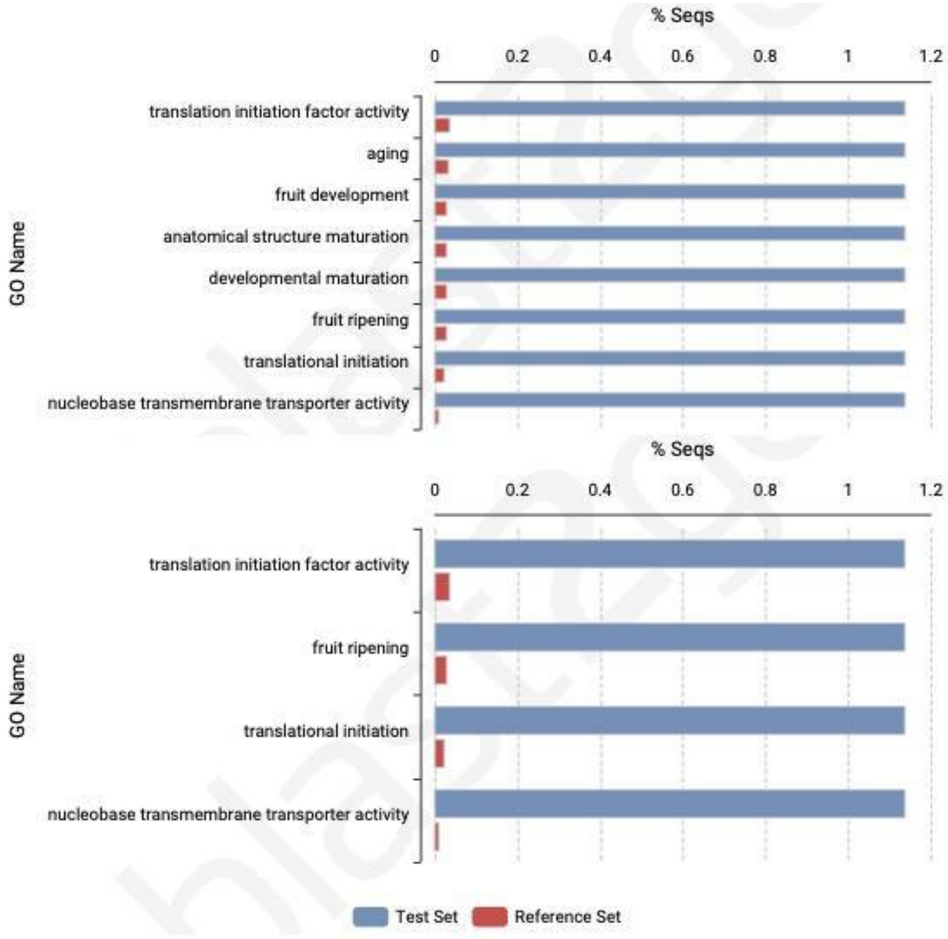

Fig. S4 The outcome of GO enrichment analysis for the pure Lowland sample (n =5), based on the set of 436 candidate genes (Table S4) detected by selective sweep mapping with SweeD. Both graphs were generated by blast2GO, and both include only significant categories as measure by a p-value of $p < 0.05$ by a Fisher's Exact Test, as corrected for multiple tests by the blast2GO program. The graphs differ in the specificity; the top graph was generated to include general terms, while the lower graph used the "reduce to most specific option" to report terms at the most specific level in the gene ontology enrichment directed acyclic graph (DAG) file.

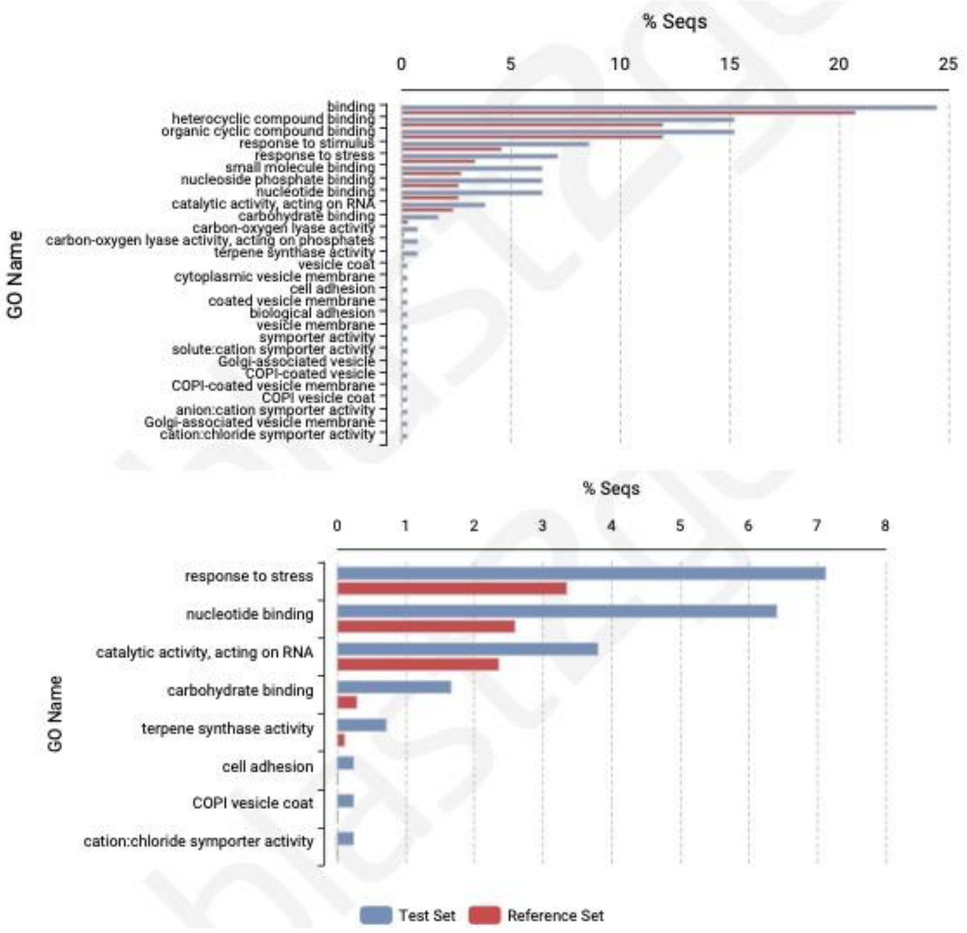

Fig. S5 The outcome of GO enrichment analysis for the pure Mexican sample (n =3), based on the set of 638 candidate genes (Table S4) detected by selective sweep mapping with SweeD. Both graphs were generated by blast2GO, and both include only significant categories as measure by a p-value of $p < 0.05$ by a Fisher's Exact Test, as corrected for multiple tests by the blast2GO program. The graphs differ in the specificity; the top graph was generated to include general terms, while the lower graph used the "reduce to most specific option" to report terms at the most specific level in the gene ontology enrichment directed acyclic graph (DAG) file.

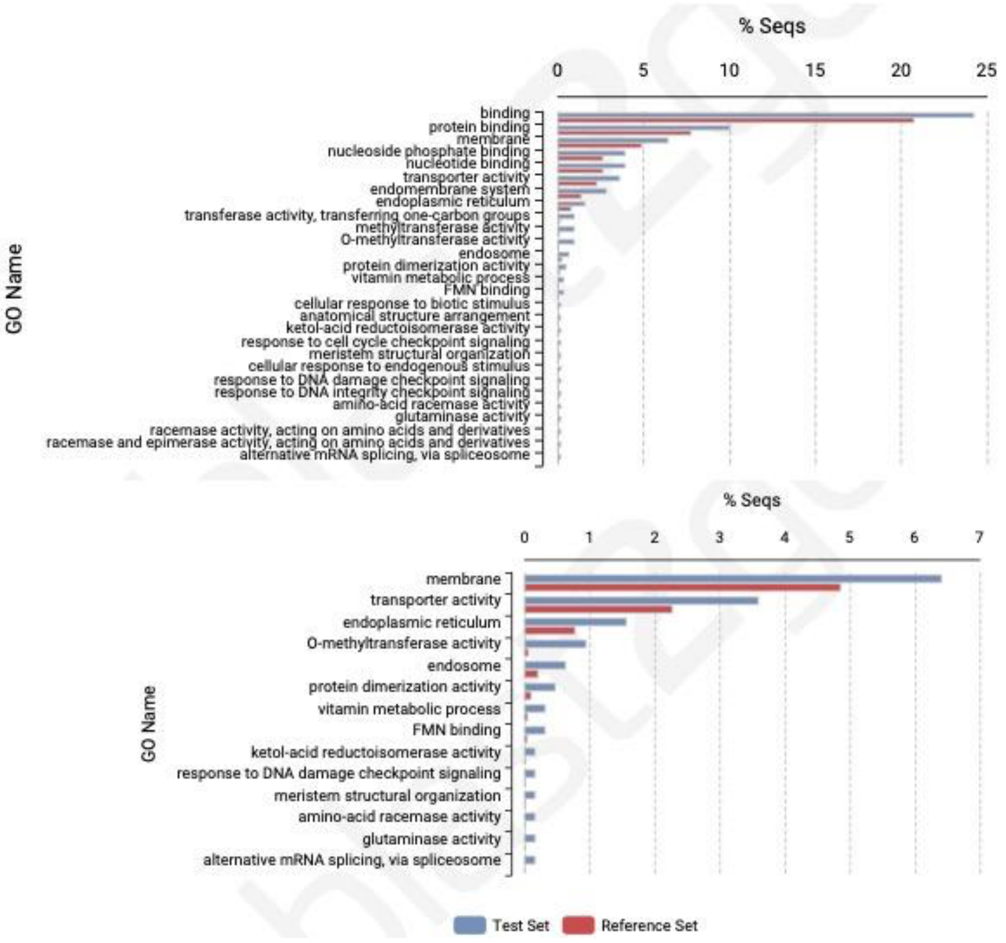

Fig. S6 Plots illustrating Fst across the 12 scaffolded pseudo-chromosomes. Each plot represents a comparison between two racial samples, based on 20kb windows along the chromosomes. In each graph, a dot represents Fst for each window, the red line represents a smoothed value along the chromosome, and the horizontal blue dotted line indicates the 1% cut-off. The three graphs are as labeled – i.e., the top graph contrasts the Mexican and Lowland sample, the middle graph contrasts the Mexican and Guatemalan sample, and the bottom graph the Lowland and Guatemalan samples.

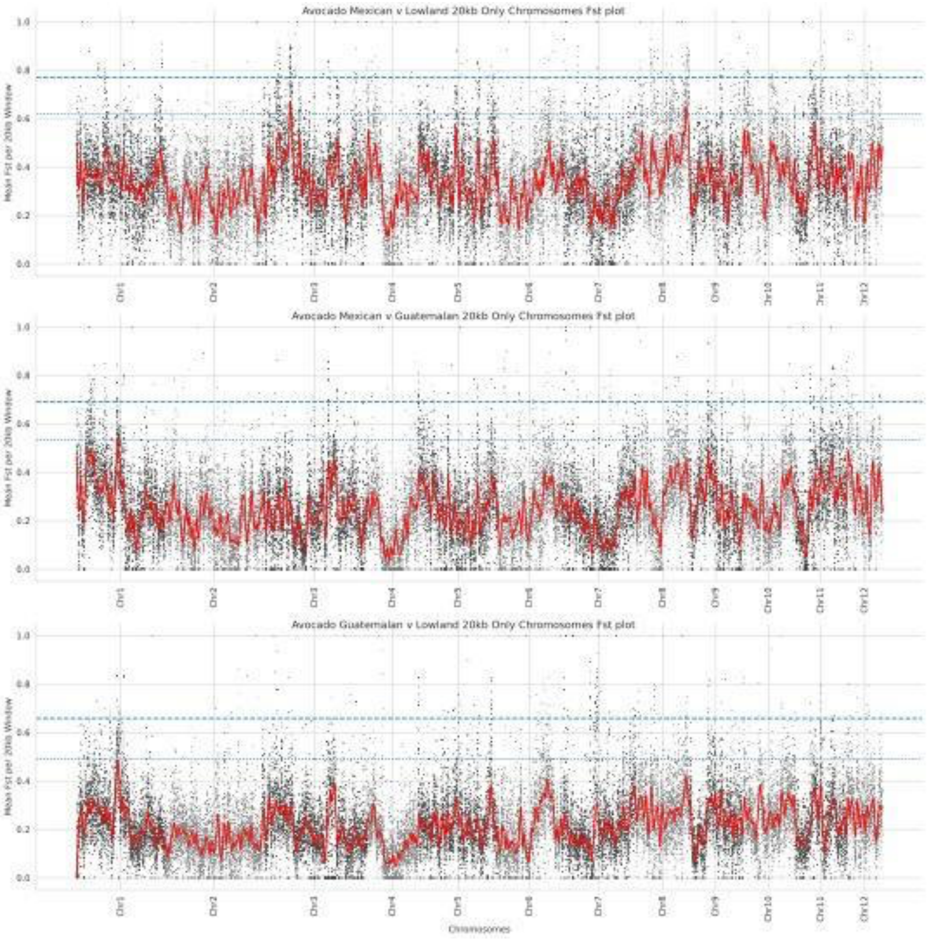

Fig. S7 The outcome of GO enrichment analysis based on the set of 396 candidate genes (Table S7) detected by Fst divergence analysis between the Mexican and Lowland pure sample. Both graphs were generated by blast2GO, and both include only significant categories as measure by a p-value of $p < 0.05$ by a Fisher's Exact Test, as corrected for multiple tests by the blast2GO program. The graphs differ in the specificity; the top graph was generated to include general terms, while the lower graph used the "reduce to most specific option" to report terms at the most specific level in the gene ontology enrichment directed acyclic graph (DAG) file.

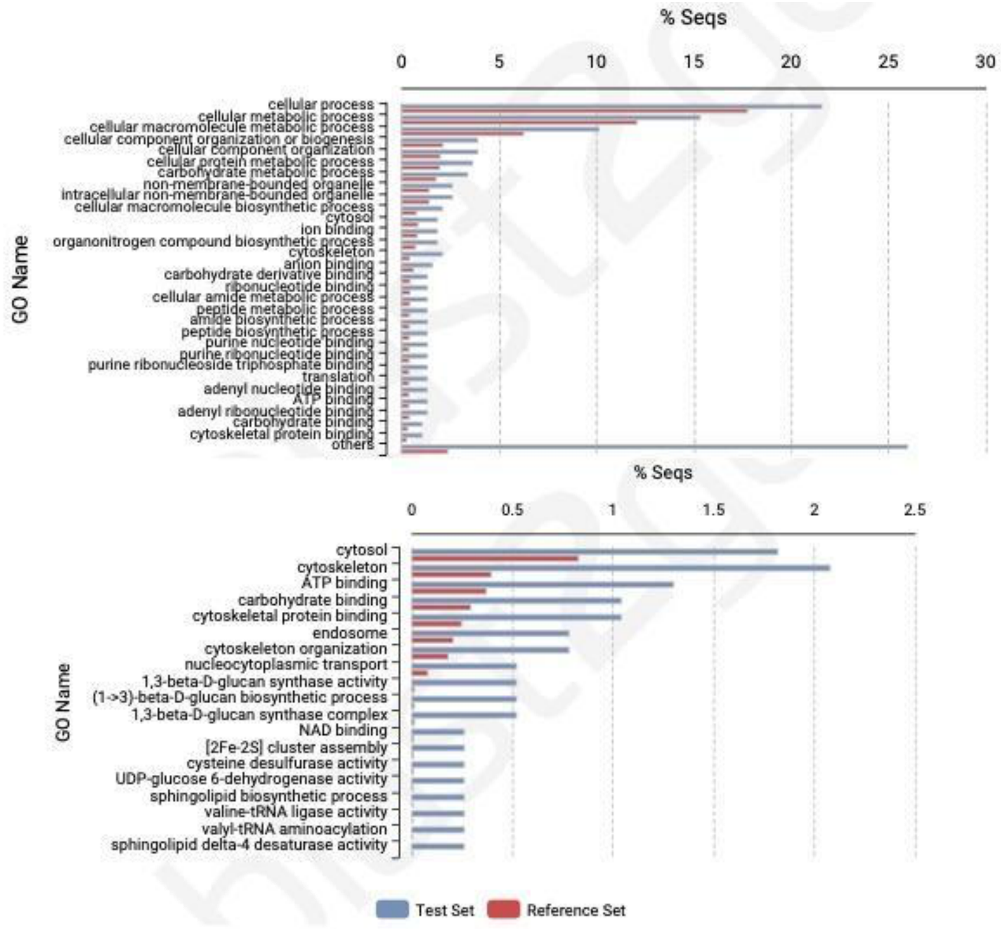

Fig. S8 The outcome of GO enrichment analysis based on the set of 387 candidate genes (Table S7) detected by Fst divergence analysis between the Mexican and Guatemalan pure samples. Both graphs were generated by blast2GO, and both include only significant categories as measure by a p-value of $p < 0.05$ by a Fisher's Exact Test, as corrected for multiple tests by the blast2GO program. The graphs differ in the specificity; the top graph was generated to include general terms, while the lower graph used the "reduce to most specific option" to report terms at the most specific level in the gene ontology enrichment directed acyclic graph (DAG) file.

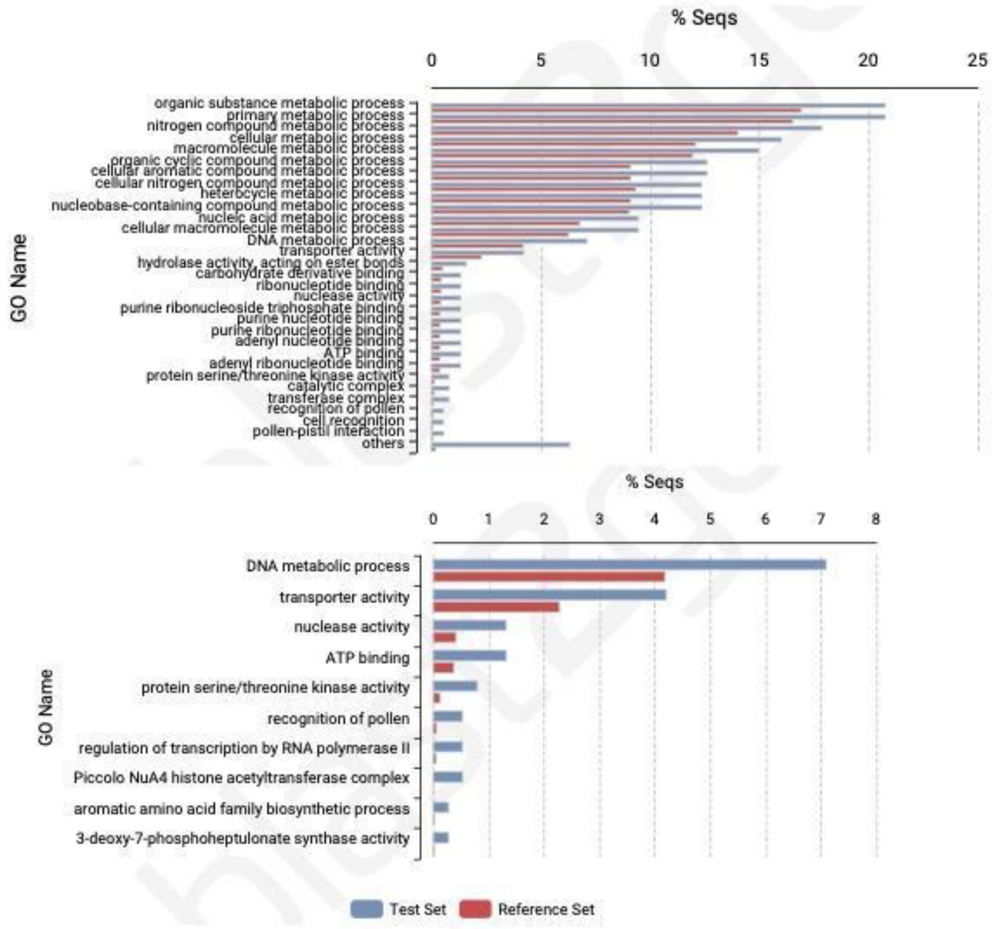

Fig. S9 The outcome of GO enrichment analysis based on the set of 384 candidate genes (Table S7) detected by *Fst* divergence analysis between the Lowland and Guatemalan pure samples. Both graphs were generated by blast2GO, and both include only significant categories as measured by a p-value of $p < 0.05$ by a Fisher's Exact Test, as corrected for multiple tests by the blast2GO program. The graphs differ in the specificity; the top graph was generated to include general terms, while the lower graph used the "reduce to most specific option" to report terms at the most specific level in the gene ontology enrichment directed acyclic graph (DAG) file.

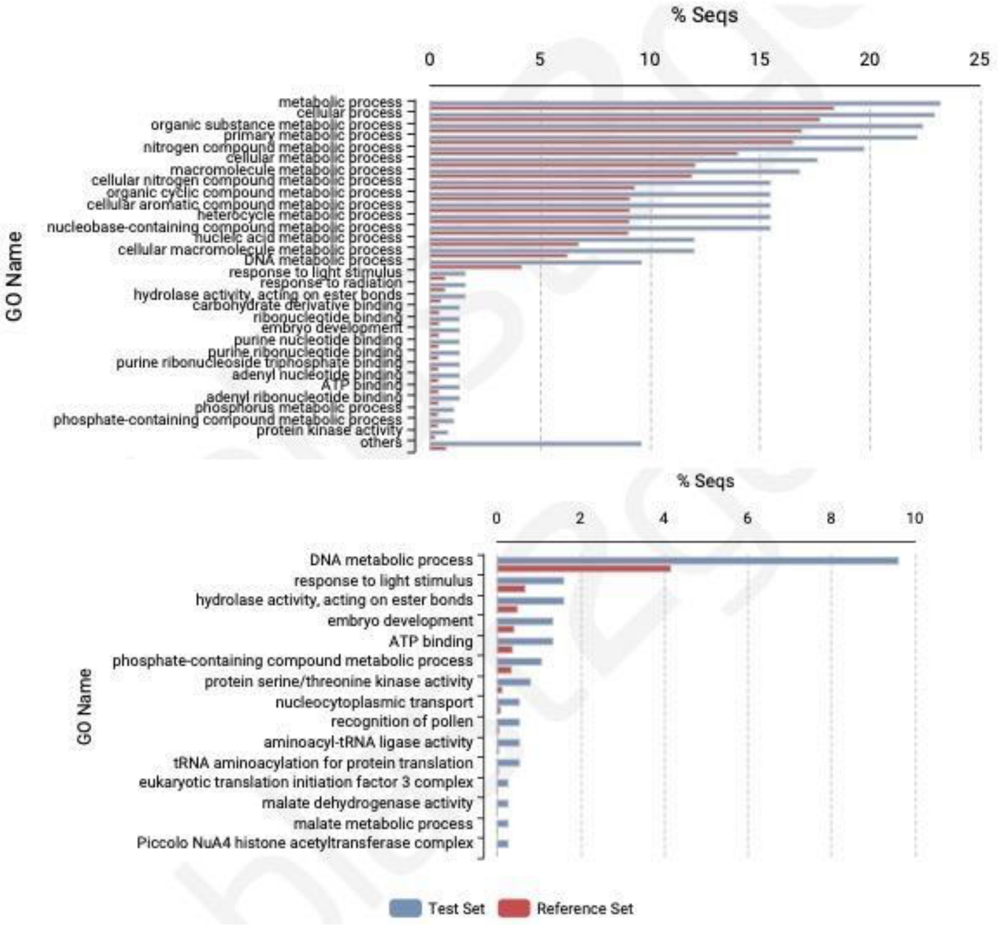

Fig. S10 The outcome of GO enrichment analysis based on the set of 466 genes (**Table S9**) detected by *Fst* divergence analysis between the A and B flowering types (Table 1). Both graphs were generated by blast2GO, and both include only significant categories as measured by a p-value of $p < 0.05$ by a Fisher's Exact Test, as corrected for multiple tests by the blast2GO program. The graphs differ in the specificity; the top graph was generated to include general terms, while the lower graph used the "reduce to most specific option" to report terms at the most specific level in the gene ontology enrichment directed acylic graph (DAG) file.

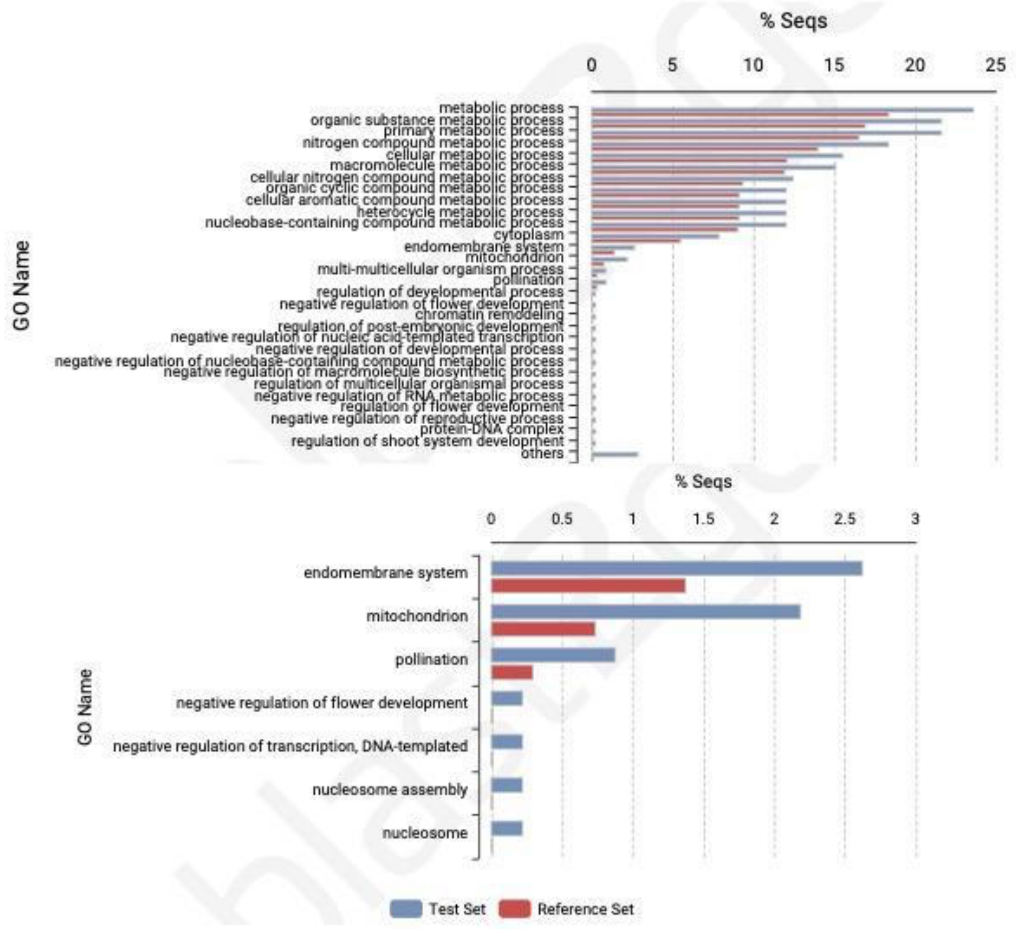

Table S1 Assembly Metrics for two Gwen assemblies, the Hass HiFi assembly, the Hass assembly and the drymifolia assembly. The Hass HiFi assembly is from (Sharma et al. 2021), and the Hass and drymifolia assemblies are from (Rendón-Anaya et al. 2019)

	**Gwen + HapSolo Contigs**	**Gwen Scaffolds**	**Hass HiFi Contigs[1]**	**Hass Contigs**	***drymifolia* Contigs**
**Assembly Size (Mb)**	1,032	703	749	913	823
**Number Fragments**	989	12	298	8,135	42,722
**Largest (kb)**	17,080	85,354	12,461	2,811	4,611
**Percent of Assembly in 12 Largest**	17.00%	78.20%	12.84%	2.74%	4.32%
**Percent in Fragments > 10Mb**	17.00%	78.20%	5.24%	0.00%	0.00%
**N50 (Mb)**	3.37	61.89	4.33	0.30	0.32
**L50**	176	5	57	770	502
**BUSCO (%)**	90.7	87.9	90.7	84.9	86.3

[1] These values differ from the original publication, based on reanalysis of the publicly available assembled genome, using the same methods we used to evaluate the Gwen genome assemblies.

Table S2 Number of biallelic SNPs and nucleotide diversity per base pair (Pi) within the pure samples of botanical races.

	Sample Size	Pi_Total [1]	Pi_Non[2]	Pi_Syn[3]
Guatemalan	10	0.00446	0.00281	0.00362
Lowland	5	0.00344	0.00214	0.00280
Mexican	3	0.00370	0.00225	0.00292

[1] Pi_Total is based on all SNPs within samples, based on mapping to the scaffolded assembly.
[2] Pi_Non is an estimate of pi to synonymous substitutions, based on non-degenerate sites in the complete gene set. Nondegenerate sites were defined in the reference and included 26,441,730 such sites.
[2] Pi_Syn is an estimate of pi for synonymous substitutions, based on four-fold degenerate sites in the complete gene set. Four-fold degenerate sites were defined in the reference and included 7,106,644 such sites.

Non-degenerate and four-fold degenerate sites were identified based on the scripts described in:

Wang, Bernhardsson and Ingvarsson. 2020. *Demography and Natural Selection Have Shaped Genetic Variation in the Widely Distributed Conifer Norway Spruce (*Picea abies*). Genome Biology and Evolution,* 12(2): 3803–3817.

Table S3 List of genes under CLR peaks, as inferred from SweeD analysis, in each of the three samples representing botanical races (see supplementary excel file TableS3)

Table S4 The outcome of enrichment analyses for comparing genes either inferred to be under selection between two races (as inferred by SweeD analyses) or identified to be under selection (SweeD) in one race and contributing to diversity (as measured by Fst) between races. Statistically significant results indicate that more genes are shared between candidate lists than expected at random.

**Comparison**[1]	**Source List 1**[2]	**Source List 2**	**No. Shared Genes**[3]	**p-value**[4]
SweeD v SweeD	Lowland	Mexican	18	**p < 0.0001**
SweeD v SweeD	Mexican	Guatemalan	2	p = 0.3692
SweeD v SweeD	Lowland	Guatemalan	0	p = 1
Fst v Sweed	Guatemalan_v_Lowland	Guatemalan	8	**p < 0.0001**
Fst v Sweed	Guatemalan_v_Lowland	Lowland	12	**p = 0.0003**
Fst v Sweed	Mexican_v_Guatemalan	Mexican	5	p = 0.6354
Fst v Sweed	Mexican_v_Guatemalan	Guatemalan	6	**p < 0.0001**
Fst v Sweed	Mexican_v_Lowland	Mexican	5	p = 0.6513
Fst v Sweed	Mexican_v_Lowland	Lowland	10	**p = 0.0028**

[1] Each comparison contrasts two lists of genes from either two SweeD analysis or from a SweeD analysis and an Fst Analysis.
[2] The list of genes from Fst analyses are denoted by, e.g., Mexican_v_Lowland, whereas genes from SweeD analyses are listed by race only.
[3] The number of genes shared between lists.
[4] The p-value, based on permutation, under the null hypothesis that the number of shared genes is random. Bolded are significant at alpha < 0.05 after multiple test correction.

Table S5 List of 20 genes under CLR peaks that are shared between races, with information from functional annotation. Only the number shared between Lowland and Mexican samples were higher than random expectation.

GeneID	Sources[1]	Protein Category[2]	GO activity[3]
Chr1.ver1.g27910	lowland, mexican	Retrovirus-related Pol polyprotein from transposon TNT 1-94 (EC 3.1.13.-)	hydrolase activity [GO:0016787]; nucleic acid binding [GO:0003676]; zinc ion binding [GO:0008270]; DNA integration [GO:0015074]
Chr10.ver1.g47660	guatemalan, mexican	Uncharacterized protein	
Chr10.ver1.g47670	guatemalan, mexican		
Chr11.ver1.g80970	lowland, mexican	Hexosyltransferase (EC 2.4.1.-)	glycosyltransferase activity [GO:0016757]
Chr12.ver1.g88360	lowland, mexican	DNA-directed RNA polymerase I subunit rpa49	nucleolus [GO:0005730]; DNA binding [GO:0003677]; DNA-directed 5'-3' RNA polymerase activity [GO:0003899]; transcription, DNA-templated [GO:0006351]
Chr12.ver1.g88370	lowland, mexican	DNA-directed RNA polymerase I subunit rpa49	nucleolus [GO:0005730]; DNA binding [GO:0003677]; DNA-directed 5'-3' RNA polymerase activity [GO:0003899]; transcription, DNA-templated [GO:0006351]
Chr2.ver1.g121020	lowland, mexican	Mediator of RNA polymerase II transcription subunit 27 isoform X1	mediator complex [GO:0016592]
Chr2.ver1.g121030	lowland, mexican		
Chr2.ver1.g131740	lowland, mexican	Mediator of RNA polymerase II transcription subunit 27 isoform X1	mediator complex [GO:0016592]
Chr3.ver1.g191700	lowland, mexican		
Chr4.ver1.g203230	lowland, mexican		
Chr4.ver1.g209590	lowland, mexican	Sugar transport protein 7 (Hexose transporter 7)	endomembrane system [GO:0012505]; integral component of membrane [GO:0016021]; plasma membrane [GO:0005886]; pollen tube [GO:0090406]; arabinose transmembrane transporter activity [GO:0042900]; symporter activity [GO:0015293]; L-arabinose transmembrane transport [GO:0042882]

Chr4.ver1.g209600	lowland, mexican	Sugar transport protein 7 (Hexose transporter 7)	endomembrane system [GO:0012505]; integral component of membrane [GO:0016021]; plasma membrane [GO:0005886]; pollen tube [GO:0090406]; arabinose transmembrane transporter activity [GO:0042900]; symporter activity [GO:0015293]; L-arabinose transmembrane transport [GO:0042882]
Chr4.ver1.g214170	lowland, mexican	WAT1-related protein	integral component of membrane [GO:0016021]; transmembrane transporter activity [GO:0022857]
Chr6.ver1.g269660	lowland, mexican		
Chr6.ver1.g269670	lowland, mexican		
Chr6.ver1.g274670	lowland, mexican	Uncharacterized protein (Fragment)	ribosome [GO:0005840]; structural constituent of ribosome [GO:0003735]; translation [GO:0006412]
Chr6.ver1.g274680	lowland, mexican	Mitogen-activated protein kinase 10-like protein	ATP binding [GO:0005524]; protein kinase activity [GO:0004672]
Chr9.ver1.g365220	lowland, mexican	RS-norcoclaurine 6-O-methyltransferase-like protein	O-methyltransferase activity [GO:0008171]; protein dimerization activity [GO:0046983]; methylation [GO:0032259]
Chr9.ver1.g365230	lowland, mexican	INO80 complex subunit B-like protein conserved region	Ino80 complex [GO:0031011]; chromatin remodeling [GO:0006338]

[1] Source refers to the samples that shared the listed gene near the CLR peak.
[2] Functional annotation from Swissprot and/or Uniref , where available.
[3] GO categories based on blast2GO analyses.

Table S6 List of genes under Fst peaks in pairwise comparisons (see supplementary excel files Table S6)

Table S7 List of the 45 genes that were identified as being under both 1% Fst peaks and under 1% CLR peaks, with functional annotation information.

GeneID	Source	Protein name2
Chr10.ver1.g43710	MvL_Low	Uncharacterized protein
Chr10.ver1.g47470	GvL_Guat	Uncharacterized protein
Chr10.ver1.g47480	GvL_Guat	Reverse transcriptase Ty1/copia-type domain-containing protein
Chr10.ver1.g47490	GvL_Guat	Reverse transcriptase Ty1/copia-type domain-containing protein
Chr11.ver1.g74150	MvL_Low	Serine/threonine-protein phosphatase 6 regulatory ankyrin repeat subunit B-like protein
Chr12.ver1.g85940	MvG_Guat	Ribonuclease HI (RNase HI) (Sto-RNase HI) (EC 3.1.26.4)
Chr12.ver1.g85950	MvG_Guat	Uncharacterized protein
Chr12.ver1.g85960	MvG_Guat	Uncharacterized protein
Chr12.ver1.g86000	MvG_Guat	Transposon Tf2-6 polyprotein (Retrotransposable element Tf2 155 kDa protein)
Chr12.ver1.g86010	MvG_Guat	
Chr12.ver1.g86020	MvG_Guat	Retrovirus-related Pol polyprotein from transposon opus [Includes: Protease (EC 3.4.23.-); Reverse transcriptase (EC 2.7.7.49); Endonuclease]
Chr12.ver1.g88570	GvL_Low	ABC transporter
Chr2.ver1.g131740	GvL_Low	Soluble starch synthase 3 chloroplastic/amyloplastic-like protein
Chr3.ver1.g164320	MvL_Mex	Putative serine/threonine protein kinase IREH1 isoform X1
Chr3.ver1.g166260	MvL_Low	
Chr3.ver1.g166280	MvL_Low	
Chr3.ver1.g179740	MvL_Low	
Chr3.ver1.g179750	MvL_Low	Transposon Tf2-6 polyprotein (Retrotransposable element Tf2 155 kDa protein)
Chr3.ver1.g193760	MvL_Mex	F-box/FBD/LRR-repeat-like protein isoform X1
Chr4.ver1.g212850	MvL_Low	Glutamate receptor 2.5-like protein isoform X1
Chr5.ver1.g216160	GvL_Low	Tetratricopeptide repeat-containing domain-containing protein
Chr5.ver1.g216190	GvL_Low	Sodium-dependent phosphate transport protein 1, chloroplastic-like protein
Chr5.ver1.g236370	MvG_Mex	Tonoplast dicarboxylate transporter (AttDT) (Sodium-dicarboxylate cotransporter-like) (AtSDAT) (Vacuolar malate transporter)
Chr5.ver1.g236380	MvG_Mex	Putative F-box protein PP2-B12
Chr5.ver1.g256990	MvL_Low	Uridine monophosphate kinase (EC 2.7.4.22) (Uridylate kinase)
Chr6.ver1.g268430	GvL_Low	
Chr6.ver1.g272410	GvL_Low	RNA-binding protein 1-like protein
Chr6.ver1.g275850	MvG_Mex	Putative leucine-rich repeat receptor-like serine/threonine-

		protein kinase
Chr6.ver1.g275860	MvG_Mex	Non-specific serine/threonine protein kinase (EC 2.7.11.1)
Chr6.ver1.g276640	MvL_Mex	DYW_deaminase domain-containing protein
Chr7.ver1.g287380	GvL_Low	Pentatricopeptide repeat-containing protein, mitochondrial
Chr7.ver1.g287390	GvL_Low	Geraniol synthase, chloroplastic
Chr7.ver1.g301160	GvL_Guat	Putative NADP-dependent oxidoreductase domain-containing protein
Chr7.ver1.g301170	GvL_Guat	Serine/threonine-protein kinase D6PK-like protein
Chr7.ver1.g301180	GvL_Guat	Serine/threonine-protein kinase D6PK-like protein
Chr7.ver1.g301190	GvL_Guat	Protein kinase domain-containing protein
Chr7.ver1.g301200	GvL_Guat	Serine/threonine-protein kinase CTR1 (EC 2.7.11.1) (Protein CONSTITUTIVE TRIPLE RESPONSE1)
Chr8.ver1.g330460	MvG_Mex	WD40 repeat
Chr8.ver1.g344340	MvL_Mex	Lipopolysaccharide core biosynthesis mannosyltransferase lpsB
Chr8.ver1.g344350	MvL_Mex	Uncharacterized protein
Chr9.ver1.g356100	GvL_Low	Retrovirus-related Pol polyprotein from transposon RE1 (Retro element 1) (AtRE1) [Includes: Protease RE1 (EC 3.4.23.-); Reverse transcriptase RE1 (EC 2.7.7.49); Endonuclease RE1]
Chr9.ver1.g356700	GvL_Low	Auxin-induced protein
Chr9.ver1.g356710	GvL_Low	Auxin-responsive protein SAUR50 (Protein SMALL AUXIN UP RNA 50)
ChrU.ver1.g471560	GvL_Low, MvL_Low	Putative LRR receptor-like serine/threonine-protein kinase
ChrU.ver1.g471570	MvL_Low	actin-related protein 2/3 complex subunit 1A-like

[1] Source refers to the comparisons used to identify candidate genes. The first three letters refer to the Fst comparson, and the last phrase refers to the Sweed analysis. E.g., MvL_Low is based on Fst genes found between Mexican (M) and Lowland (L) races and the comparison of those genes to the candidate gene set from Lowland (Low) CLR analyses.
[2] Functional annotation from Swissprot and/or UniRef, where available.

Table S8 List of 466 genes that were under Fst peaks when comparing A flowering types to B flowering types and that were the basis for GO enrichment analysis (Figure S10) (see supplementary excel file Table S8).

Methods S1 For demographic analyses, the mappability mask was made with (http://lh3lh3.users.sourceforge.net/snpable.shtml) by generating 150 bp mers in 1 bp increments across the genome, then mapping sequences back to the genome with BWA v0.7.8-r455 (Li and Durbin 2010) and identifying "mappable" regions where the majority of sequences mapped uniquely without mismatches. To include only regions with sufficient sequencing coverage, we calculated the coverage from the alignment file of each sample with the bedcov program from samtools (v1.10); we then created a mask per sample that only included regions with sequencing coverage higher than 5x.
